# Cascade Electrocatalytic Reduction of Nitrate to Ammonia Using Bimetallic Covalent Organic Frameworks with Tandem Active Sites

**DOI:** 10.1002/anie.202507956

**Published:** 2025-06-18

**Authors:** Jian Zhong, Haiyan Duan, Mingquan Cai, Ying Zhu, Zhenlin Wang, Xingchi Li, Zhengliang Zhang, Wenqiang Qu, Kai Zhang, Donglin Han, Danhong Cheng, Yongjie Shen, Ming Xie, Emiliano Cortes, Dengsong Zhang

**Affiliations:** ^1^ International Joint Laboratory of Catalytic Chemistry State Key Laboratory of Advanced Special Steel Innovation Institute of Carbon Neutrality Department of Chemistry College of Sciences Shanghai University Shanghai 200444 People's Republic of China; ^2^ Nanoinstitute Munich Faculty of Physics Ludwig‐Maximilians‐Universität (LMU) Munich 80539 Germany; ^3^ Department of Chemistry University of Toronto 80 St. George Street Toronto ON M5S 3H6 Canada; ^4^ Institute for Chemical Reaction Design and Discovery (WPI‐ICReDD) Hokkaido University Sapporo 001‐0021 Japan; ^5^ Department of Chemical Engineering University of Bath Bath BA2 7AY UK

**Keywords:** Ammonia synthesis, Cascade electrocatalysis, Covalent organic frameworks, Nitrate reduction, Tandem active sites

## Abstract

Electrochemical nitrate reduction reaction (NO_3_RR) is a promising approach to simultaneously realize pollutant removal and ammonia generation. However, this process involves the transfer of eight electrons and nine protons along with multiple by‐products, resulting in a significant challenge for achieving high ammonia yield and selectivity. Herein, we introduced bimetallic covalent organic frameworks catalysts with Cu and Co active sites to achieve a two‐step tandem reaction, avoiding excessive nitrite accumulation and enabling efficient NO_3_RR. For the initial two‐electron process, the Cu sites in the bimetallic catalyst exhibit a strong binding affinity with nitrate, promoting their conversion to nitrite. The Co sites enhance the supply and adsorption of active hydrogen and stabilize the subsequent six‐electron process, thereby improving the overall catalytic efficiency. Compared to monometallic Cu and Co catalysts, the CuCo bimetallic catalyst demonstrates superior ammonia yield and Faradaic efficiency (NH_3_ yield rate = 20.8 mg·h^−1^·cm^−2^, FE = 92.16% in 0.3 M nitrate). Such coordinated two‐step process advances the efficiency and applicability of NO_3_RR through optimizing a cascade catalytic reaction, thereby establishing an innovative path for the engineering of NO_3_RR electrocatalysts.

## Graphical abstract

Fabricating covalent organic frameworks electrocatalyst with bimetallic active sites and promoting cascade electrocatalytic reduction of nitrate to ammonia through the copper‐cobalt tandem mechanism. The Cu sites serve as the activation centers for adsorption and the initial 2e^−^ process. The ‐N‐Co‐O‐ interface promotes water adsorption and dissociation, facilitates the formation of *H‐rich interface, and boosts the subsequent 6e^−^ process, thus accelerating efficient synthesis of ammonia.

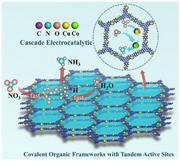

## Introduction

Ammonia (NH_3_) remains not only an essential precursor in chemical industries^[^ ^1^, ^2^, ^3^, ^4^ ^]^ but also a next‐generation hydrogen storage medium and carbon‐neutral energy carrier.^[^ ^5^ ^]^ However, the conventional Haber‐Bosch process for industrial NH_3_ synthesis involves high‐energy consumption and generates significant greenhouse gas emissions.^[^ ^6^ ^]^ As an attractive alternative, electrocatalytic nitrate reduction reaction (NO_3_RR) for NH_3_ synthesis has emerged due to its environmental friendliness and mild reaction conditions.^[^ ^7^, ^8^, ^9^ ^]^ Alternative to nitrogen and nitric oxide,^[^ ^9^, ^10^, ^11^ ^]^ NO_3_^−^ benefits from its lower dissociation energy, higher solubility and rapid reduction kinetics at the liquid‐solid interface, thus being regarded as a more promising N source for NH_3_ production.^[^ ^7^, ^12^, ^13^, ^14^, ^15^ ^]^ Additionally, NO_3_^−^ is a prevalent harmful pollutant in contaminated groundwater, industrial wastewater and nuclear emissions, which poses risks to both the environment and human health. Consequently, converting nitrate into the value‐added product NH_3_ is environmentally beneficial and economically advantageous. The NO_3_RR process (NO_3_^−^ + 8e^−^ + 9H^+^ → NH_3_ + 3H_2_O, E^0^ = 0.69 V vs. RHE) involves a complicated eight‐electron and nice‐proton transfer process,^[^ ^16^, ^17^, ^18^, ^19^ ^]^ and is prone to generate by‐product (NO_2_^−^, NO, NH_2_OH, N_2_H_4_, etc.).^[^ ^20^, ^21^, ^22^, ^23^, ^24^ ^]^ This complex process can be divided into two consecutive reactions: a 2e^−^ process from NO_3_^−^ to NO_2_^−^ and a 6e^−^ process from NO_2_^−^ to NH_3_.^[^ ^25^, ^26^, ^27^ ^]^ Matching the reactivity of two‐step reactions is crucial to achieve high ammonia yield and Faradaic efficiency (FE).

Early studies have shown that Cu could effectively facilitate the adsorption of NO_3_^−^ due to the similarity of energy levels between the d orbitals of Cu and the LUMO of nitrate.^[^ ^28^, ^29^, ^30^, ^31^ ^]^ Additionally, the low hydrogen accumulation on the Cu^[^ ^32^, ^33^, ^34^ ^]^ surface promotes NO_3_^−^ reduction while suppressing hydrogen evolution reaction (HER).^[^ ^35^, ^36^ ^]^ However, insufficient *H supply may kinetically limit the hydrogenation step in the NO_3_RR.^[^ ^37^, ^38^ ^]^ This calls for the rational regulation of water adsorption and enhanced supply of *H ^[^ ^39^, ^40^ ^]^ over the surface of Cu‐based catalyst.^[^ ^41^, ^42^ ^]^ The inherent oxyphilic nature and electronegativity of Co could facilitate *H adsorption and supply,^[^ ^43^, ^44^, ^45^, ^46^, ^47^ ^]^ creating an excellent interfacial environment for the NO_3_^−^ hydrogenation process while mitigating the formation of by‐products. Therefore, coupling the Cu and Co active sites has significant potential to optimize the deoxygenation and hydrogenation steps, thus matching the two‐step reactions and promoting tandem NO_3_RR process.^[^ ^48^, ^49^, ^50^ ^]^ Recently, covalent organic frameworks (COFs) have garnered significant attention due to their high crystallinity,^[^ ^51^, ^52^, ^53^ ^]^ stable structure,^[^ ^54^, ^55^ ^]^ abundant pore channels,^[^ ^56^, ^57^, ^58^ ^]^ modifiable frameworks,^[^ ^53^, ^59^, ^60^, ^61^ ^]^ and fully exposed metal active sites,^[^ ^62^, ^63^, ^64^, ^65^ ^]^ extensively exploring as promising electrocatalysts for targeted catalytic applications. Hydrazone‐linked COFs represent a promising platform for forming new metal coordination sites containing both N and O atoms after synthesis process, thereby ensuring robust stabilization of metal centers.^[^ ^66^, ^67^ ^]^ Therefore, it can be envisioned that the chemical designability and tunability of hydrazone‐linked COFs can not only provide a promising platform for manipulating the NO_3_RR process at the electrocatalytic interface, but also contribute to elucidating the cascade electrocatalytic mechanism and reaction pathway, as well as understanding the NO_3_RR catalytic process to improve ammonia yield and FE. However, the research in this regard is scarcely reported.

Herein, we introduced COFs electrocatalyst with bimetallic active sites to match the two‐step cascade reaction, thus avoiding excessive nitrite accumulation and enabling an efficient NO_3_RR process through Cu‐Co tandem mechanism. Focusing on the initial two‐electron process, the Cu sites in the bimetallic catalyst exhibit a strong binding affinity with nitrate and promote their efficient conversion to nitrite. The Co sites enhance the supply and adsorption of active hydrogen during the later six‐electron process, thereby improving the overall catalytic efficiency. Consequently, the CuCo bimetallic catalyst demonstrates superior ammonia yield and FE (NH_3_ yield rate = 20.8 mg·h^−1^·cm^−2^, FE = 92.16% in 0.3 M nitrate), much higher than those of monometallic Cu and Co catalysts. Such a highly matched two‐step process provides a feasible strategy for efficiently coupling the cascade process, thereby advances the efficiency and applicability of NO_3_RR.

## Results and Discussion

**Figure 1:**
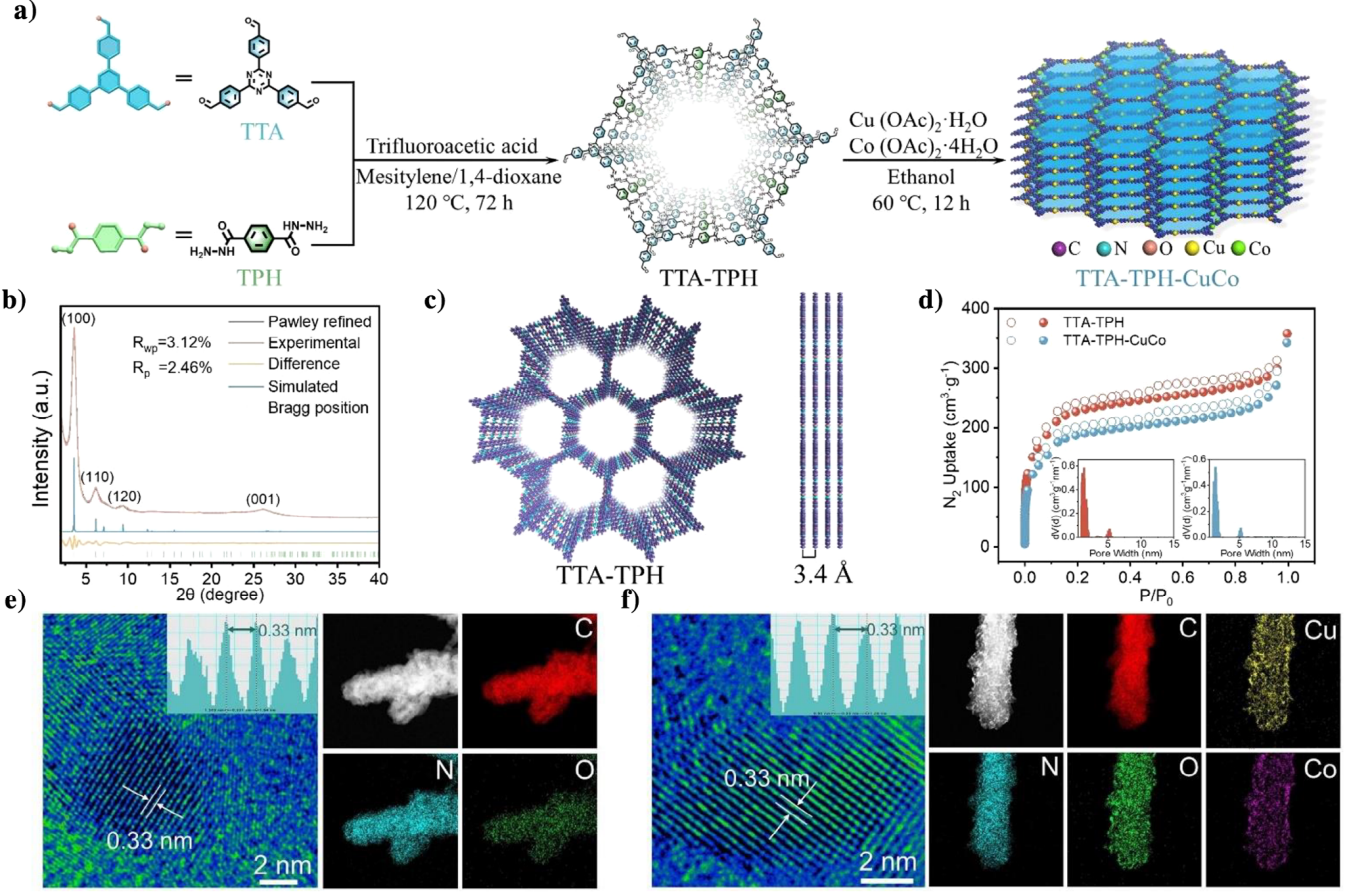
Synthesis process and characterizations of catalysts. a) The schematic diagram of preparing TTA‐TPH and TTA‐TPH‐CuCo. b) PXRD patterns and AA stacking simulation of TTA‐TPH. c) Top and side views of the refined AA model for TTA‐TPH. d) Nitrogen adsorption‐desorption isotherms and pore size distributions (inset) of TTA‐TPH and TTA‐TPH‐CuCo. (e, f) HR‐TEM images and EDS mapping images of TTA‐TPH and TTA‐TPH‐CuCo, inset is the distance of (001) lattice plane.

A hydrazone‐linked COFs catalyst with nitrogen‐oxygen heteroatom coordination sites was successfully synthesized via a Schiff base reaction using a solvothermal method. As shown in Figure 1a, 4,4′,4″‐ (1,3,5‐triazine‐2,4,6‐triyl) tribenzaldehyde (TTA) and terephthalohydrazide (TPH) were used as monomers. The solvent ratio was optimized (mesitylene: 1,4‐dioxane = 3:7), and the reaction was carried out at 120 °C for 72 h to synthesize TTA‐TPH. Then, TTA‐TPH was dispersed in ethanol containing different concentrations of Cu ions, Co ions, or a mixture of Cu and Co ions. Subsequently, the hydrazone bonds of TTA‐TPH could effectively coordinate with metal ions under a nitrogen atmosphere at 60 °C, yielding a series of crystalline hydrazone‐linked COFs: TTA‐TPH‐Cu, TTA‐TPH‐Co and TTA‐TPH‐CuCo.

The Powder X‐ray diffraction (PXRD) and nitrogen adsorption‐desorption measurements were performed on TTA‐TPH and TTA‐TPH‐CuCo to analyze their crystallinity and porous characteristics.^[^ ^67^ ^]^ As shown in Figure 1b, four distinct diffraction peaks were clearly observed at 3.58°, 6.13°, 9.38° and 26.21°, corresponding to the (100), (110), (120) and (001) crystal planes, respectively. The PXRD experimental data of TTA‐TPH were subjected to Pawley refinement, which providing good refinement values (R_wp _= 3.12% and R_p _= 2.46%). Besides, the experimental results showed good agreement with the simulated AA stacking model (Figure 1c and Table S1), which possessed the *P*6 space group with unit cell parameters: a  =  b  =  28.98 Å, c  =  3.37 Å, α = β = 90°, γ = 120°. Then, the nitrogen adsorption‐desorption measurements were conducted to investigate the specific surface area, and the values of TTA‐TPH and TTA‐TPH‐CuCo were calculated to be 844 and 699 m^2^·g^−1^, respectively (Figure 1d). The inset in Figure 1d shows that the pore size distribution of TTA‐TPH and TTA‐TPH‐CuCo both located around 1.36 nm, which is in line with the simulated AA model.

The morphology of TTA‐TPH‐CuCo was carefully studied using scanning electron microscopy (SEM), transmission electron microscopy (TEM), high‐resolution TEM (HR‐TEM),^[^ ^66^ ^]^ and atomic force microscopy (AFM). It could be clearly observed that the TTA‐TPH‐CuCo demonstrate a layered stacking morphology with an average thickness of approximately 5∼10 nm (Figures S1 and S2). The favorable nanoscale dimensions and large surface area are beneficial for the full exposure of active sites. As shown in Figure 1e,f, HR‐TEM of TTA‐TPH and TTA‐TPH‐CuCo both exhibited evident lattice fringes of (001) plane with a distance of 0.33 nm and long‐range ordered channels, indicating the intactness of porous and ordered morphology before and after metallization. This aligns well with the PXRD and AA model simulations. Furthermore, the TEM and HR‐TEM revealed no metallic clusters (Figure S3), indicating the absence of aggregated metal clusters due to the highly ordered structure and uniformly dispersed hydrazine‐bond coordination sites. Energy‐dispersive X‐ray spectroscopy (EDS) revealed a uniform distribution of C, N and O throughout the entire TTA‐TPH framework. It could be clearly observed that the Cu and Co elements were evenly distributed on the metallized TTA‐TPH‐CuCo framework (Figure S4), providing significant possibility for NO_3_RR with high activity.

**Figure 2:**
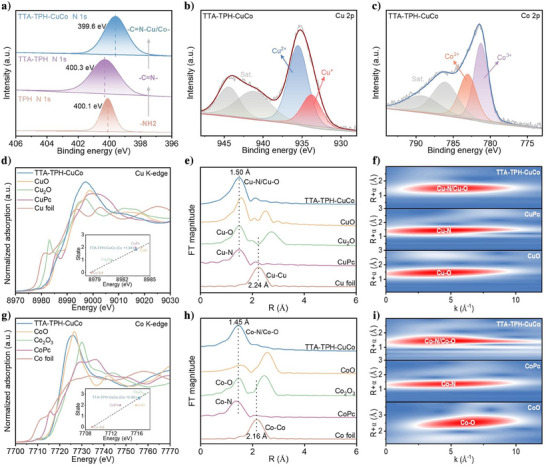
Structural characterizations of catalysts. a) XPS N1s spectra of TPH monomer, TTA‐TPH and TTA‐TPH‐CuCo. b) XPS Cu 2p spectra and c) XPS Co 2p spectra of TTA‐TPH‐CuCo. d) Cu K‐edge XANES of TTA‐TPH‐CuCo and its valence state fitting calibration curve (inset). e) FT‐EXAFS spectra of Cu for TTA‐TPH‐CuCo, CuO, Cu_2_O, Cu phthalocyanine (Pc) and Cu foil. f) WT‐EXAFS plots of Cu for CuO, CuPc, and TTA‐TPH‐CuCo. g) Co K‐edge XANES of TTA‐TPH‐CuCo and its valence state fitting calibration curve (inset). h) FT‐EXAFS spectra of Co for TTA‐TPH‐CuCo, CoO, Co_2_O_3_, CoPc, and Co foil. i) WT‐EXAFS plots of Co for CoO, CoPc and TTA‐TPH‐CuCo.

We used Fourier‐transform infrared spectroscopy (FT‐IR), ^13^C solid‐state nuclear magnetic resonance (NMR) and X‐ray photoelectron spectroscopy (XPS) to verify the chemical structure of TTA‐TPH and TTA‐TPH‐CuCo. Firstly, FT‐IR spectra of TTA‐TPH and TTA‐TPH‐CuCo both display a strong C = N stretching vibration band at 1628 cm^−1^,^[^ ^68^, ^69^, ^70^ ^]^ confirming the successful formation of hydrazone bonds (Figure S5). Additionally, no significant changes were observed in the FT‐IR spectra after metal coordination, indicating successful metalation without altering the framework structure. The characteristic NMR signals for the C = N and C = O bonds further confirm the formation of hydrazone bonds in our COF (Figure S6). Furthermore, the XPS full spectra of TTA‐TPH‐Cu, TTA‐TPH‐Co, and TTA‐TPH‐CuCo demonstrated the presence of C, N, O, Cu, and Co elements in their frameworks (Figure S7). Compared with XPS N1s of TPH monomer in Figure 2a, a new peak located at 400.3 eV corresponds to ‐C = N‐ bonds were observed, further confirming the formation of TTA‐TPH‐CuCo. In addition, the XPS N1s spectra of COFs coordinated with metals shifted to lower binding energies, which is likely attributable to the successful formation of metal‐N bond (Figure S8). The Cu 2p spectrum of TTA‐TPH‐CuCo exhibits two characteristic peaks at binding energies of 935.5  and 933.4 eV,^[^ ^28^, ^71^ ^]^ which can be assigned to Cu^2+^ and Cu^+^ species, respectively (Figure 2b). Similarly, the Co 2p spectrum displays distinct peaks at 781.2  and 782.8 eV,^[^ ^43^ ^]^ corresponding to Co^3+^ and Co^2+^ oxidation states (Figure 2c). Notably, the binding energy positions of Cu 2p and Co 2p orbitals for TTA‐TPH‐CuCo reveals no observable shifts compared with TTA‐TPH‐Cu and TTA‐TPH‐Co (Figure S9). This indicates that both Cu and Co maintain their original electronic environments through coordinating with electron‐withdrawing groups (‐C = N and ‐C = O) in the COFs, effectively suppressing the formation of metallic clusters.

X‐ray absorption near‐edge structure (XANES) were further employed to clarify the chemical states and local coordination environments of Cu and Co in TTA‐TPH‐CuCo. As shown in the Cu K‐edge XANES spectrum (Figure 2d), the absorption edge of TTA‐TPH‐CuCo is positioned between those of CuO (Cu^2+^) and Cu_2_O (Cu^+^) reference compounds, unambiguously demonstrating the coexistence of mixed Cu^2+^/Cu^+^ oxidation states. Analogously, the Co K‐edge XANES spectrum (Figure 2g) reveals an absorption edge energy situated between those of CoO (Co^2+^) and Co_2_O_3_ (Co^3+^), providing definitive evidence for the simultaneous presence of Co^2+^ and Co^3+^ species. This remarkable agreement between XANES and XPS results confirms the presence of bimetallic centers with multivalent characteristics in the TPH‐CuCo system. Moreover, the valence state fitting curves demonstrate that the average oxidation states of Cu and Co atoms in TTA‐TPH‐CuCo are 1.84 and 2.65, respectively (inset of Figure 2d,g). The Fourier‐transformed extended X‐ray absorption fine structure (FT‐EXAFS) analysis revealed prominent coordination peaks at 1.50 Å for Cu and 1.45 Å for Co, corresponding to metal‐ligand bonding distances characteristic of Cu‐N/Cu‐O and Co‐N/Co‐O coordination environments, respectively. As shown in Figure 2e,h, the spectra did not exhibit the metal‐metal bonding features typically observed at 2.24 Å (Cu‐Cu) and 2.16 Å (Co‐Co), which eliminate the possibility of forming metallic clusters or nanoparticles on the catalyst surface. The EXAFS fitting results show that the coordination numbers of Cu and Co atoms in TTA‐TPH‐CuCo are 2.95 and 3.91 (Figures S10–S15 and Tables S2, S3), respectively. The wavelet transform (WT)‐EXAFS more clearly demonstrates the presence of Cu‐N/Cu‐O and Co‐N/Co‐O scattering paths in TTA‐TPH‐CuCo (Figure 2f,i).^[^ ^72^, ^73^, ^74^ ^]^ Integrating the above‐mentioned XANES results with the XPS spectra, we could conclude that Cu and Co species are chemically anchored within the TTA‐TPH framework through covalent bonding with N and O heteroatoms. Overall, the above structural characterizations confirm that TTA‐TPH‐CuCo are efficiently fabricated with superior crystallinity, high porosity while enriching the Cu and Co active sites.

**Figure 3:**
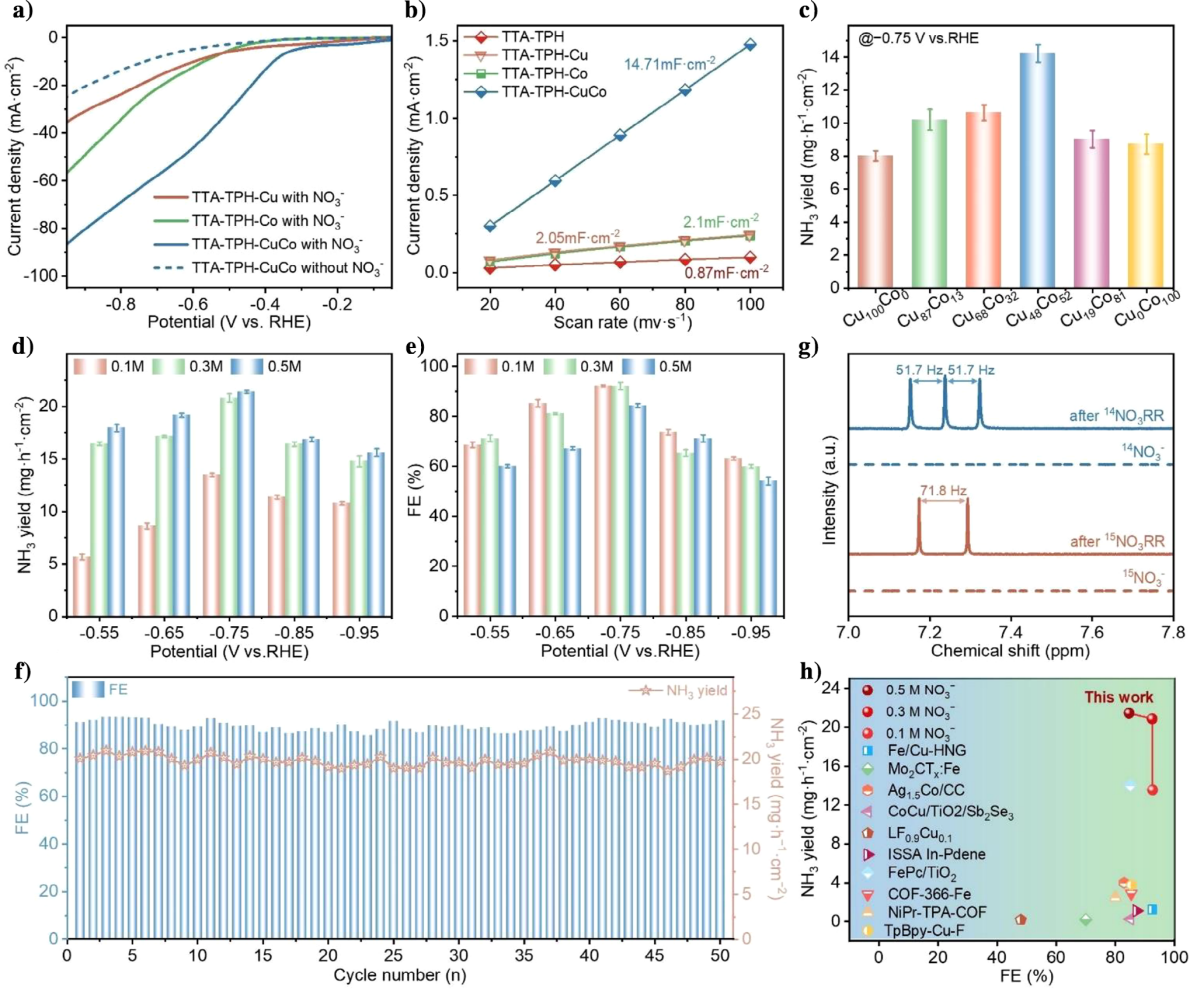
NO_3_RR performance. a) LSV curves in 0.5 M K_2_SO_4_ with and without 0.1 M NO_3_^−^. b) The C*_dl_* comparison of all samples. c) NH_3_ yield rate of TTA‐TPH‐CuCo with different CuCo proportions. d) NH_3_ yield and e) FE of TTA‐TPH‐CuCo under different potentials and NO_3_^−^ concentrations. f) The NH_3_ yield and FE of TTA‐TPH‐CuCo at −0.75 V versus RHE during fifty cycling tests. g) ^1^H NMR spectra of TTA‐TPH‐CuCo in ^15^NO_3_^−^ and ^14^NO_3_^−^ electrolytes before and after NO_3_RR. h) Comparison of NH_3_ yield and FE of TTA‐TPH‐CuCo with recently reported NO_3_RR electrocatalysts (see Supporting Information for detailed references). (Error bars in 3c–e correspond to the standard deviations of three measurements, the center value of error bars is the average of three measurements).

We then conducted NO_3_RR tests at room temperature in an H‐cell containing a neutral electrolyte (0.5 M K_2_SO_4_) with different concentrations of KNO_3_ (0.1 , 0.3 and 0.5 M) to evaluate the electrocatalytic performance. As shown in Figures 3a and S16, the linear sweep voltammetry (LSV) experiments were conducted in 0.5 M K_2_SO_4_ solution with and without 0.1 M NO_3_^−^ (from 0.05  to −0.95 V vs. RHE). Firstly, the TTA‐TPH‐CuCo exhibits a significantly higher current density upon adding NO_3_^−^ into the system, indicating the high NO_3_RR activity. Compared to the monometallic TTA‐TPH‐Cu and TTA‐TPH‐Co, the bimetallic TTA‐TPH‐CuCo shows a more positive onset potential, suggesting that the reaction could take place at a relatively positive potential, thereby reducing the energy required to initiate the reaction. Moreover, within the same potential range, TTA‐TPH‐CuCo displays a higher current density, indicating its ability to drive a faster NO_3_RR rate, thereby reducing more nitrate per unit time. The double‐layer capacitances (C*_dl_*) could be calculated based on cyclic voltammetry (CV) curves and evaluate the electrochemical active surface area (ECSA) of our catalysts,^[^ ^75^, ^76^ ^]^ thus further accessing their intrinsic catalytic activity (Figure S17). As depicted in Figure 3b, the C*_dl_* value of TTA‐TPH‐CuCo was calculated to be 14.71 mF·cm^−2^, significantly higher than that of TTA‐TPH (0.87 mF·cm^−2^), TTA‐TPH‐Cu (2.05 mF·cm^−2^), and TTA‐TPH‐Co (2.1 mF·cm^−2^). The larger ECSA of TTA‐TPH‐CuCo indicates that more catalytic active sites are exposed to the electrolyte, allowing much effective interaction with NO_3_^−^ and facilitating rapid mass transfer processes, thereby accelerating the NO_3_RR reaction rate. We further calculated the turnover frequency (TOF) of TTA‐TPH‐Cu, TTA‐TPH‐Co, and TTA‐TPH‐CuCo catalysts, which were 4152, 5193, and 7882 h^−1^, respectively, under identical conditions (Figure S18). This indicates that TTA‐TPH‐CuCo catalyst achieves significantly enhanced reaction throughput per active site per unit time, confirming its superior intrinsic activity per catalytic center.

We then probed the effect of Cu/Co ratios in detail in TTA‐TPH‐CuCo on NO_3_RR performance and determined their actual metal content using inductively coupled plasma optical emission spectroscopy (Figure S19). A series of catalysts with different Cu/Co ratios were subjected to NO_3_RR measurements at −0.75 V versus RHE. We quantitatively determined ammonia concentration using UV‐vis absorption spectroscopy, with the corresponding calibration curve presented in Figure S20. As displayed in Figure 3c, TTA‐TPH‐Cu_48_Co_52_ exhibited exceptional performance, achieving an NH_3_ yield rate of 14.22 mg·h^−1^·cm^−2^ and an FE of 90.48% (Figure S21) in 0.5 M K_2_SO_4_ containing 0.1 M NO_3_^−^ (unless otherwise specified, TTA‐TPH‐Cu_48_Co_52_ will be used for all subsequent experiments and denoted as TTA‐TPH‐CuCo in the context of this study). We then quantitatively detected the NO_2_^−^ during NO_3_RR process using ion chromatograph. The TTA‐TPH‐Cu catalyst exhibited substantial NO_2_^−^ accumulation (87.05 ppm), while TTA‐TPH‐CuCo showed an 80.84% reduction in nitrite accumulation (16.68 ppm), reaching similar levels to TTA‐TPH‐Co (Figure S22). This demonstrates cobalt incorporation significantly enhances nitrite conversion efficiency. Moreover, no hydroxylamine (NH_2_OH) was detected during the NO_3_RR process (Figure S23). We also quantitatively analyzed the hydrogen produced during the NO_3_RR process using gas chromatography (Figure S24). The results revealed that TTA‐TPH‐CuCo exhibited significantly lower FE for hydrogen production (FE = 3.8%) compared to TTA‐TPH‐Cu (FE = 17.3%) and TTA‐TPH‐Co (FE = 9.4%), suggesting enhanced *H utilization for the *NO hydrogenation on this catalyst. In the NO_3_RR process, Cu dominates the first‐step conversion of NO_3_^−^ to NO_2_^−^, generating a large amount of NO_2_^−^. However, the insufficient *H coverage on Cu surface results in kinetic limitations, where the limited *H adsorption capacity fails to sustain the subsequent hydrogeneration rate required for NO_2_^−^ conversion. This kinetic mismatch leads to significant NO_2_^−^ accumulation, ultimately compromising the NH_3_‐FE. In contrast, Co demonstrates exceptional catalytic performance in the critical second‐stage conversion of NO_2_^−^ to NH_3_. Comparative analysis reveals that TTA‐TPH‐Co achieves a 91% reduction in NO_2_^−^ yield and 27% enhancement in NH_3_‐FE compared to TTA‐TPH‐Cu, demonstrating Co's superior capability in preventing NO_2_^−^ accumulation. Therefore, by adjusting the composition and distribution of Cu and Co active sites, TTA‐TPH‐CuCo achieves synergistic tandem catalysis, where Cu efficiently drives the reduction of NO_3_^−^ to NO_2_^−^ while Co ensures rapid NO_2_^−^ conversion. This precisely engineered coordination between successive reaction steps enables TTA‐TPH‐CuCo to attain remarkable NH_3_ yields of 14.22 mg·h^−1^·cm^−2^ with FE values of 90.5 %, representing a significant performance improvement over single‐metal based catalysts TTA‐TPH‐Cu (NH_3_ yield = 8.08 mg·h^−1^·cm^−2^, FE = 64.5%) and TTA‐TPH‐Co (NH_3_ yield = 8.70 mg·h^−1^·cm^−2^, FE = 81.2%). The excellent NO_3_RR performance of TTA‐TPH‐CuCo surpasses that of most reported catalysts (Figure 3h and Table S4).

The NO_3_RR performance is strongly influenced by the local chemical environment. In situ pH monitoring reveals minimal pH variation during NO_3_^−^ adsorption due to mass transfer limitations, followed by a rapid pH surge triggered by accumulated OH^−^ (Figure S25). The competing NO_3_RR and HER processes exhibit pH‐dependent behavior, where the alkaline conditions favor NO_3_RR kinetics while suppressing HER, thereby enhancing *H utilization efficiency and overall NO_3_RR performance.

We further conducted the NO_3_RR measurement over TTA‐TPH‐CuCo catalysts at different potential. As shown in Figure 3d,e, the NH_3_ yield and FE over the TTA‐TPH‐CuCo displays a volcano‐shaped curve from −0.55 to −0.95 V, and achieving highest NH_3_ yield of 20.8 mg·h^−1^·cm^−2^ and FE of 92.16% in 0.3 M nitrate at −0.75 V. When increasing the applied cathodic potential from −0.55 to −0.75 V versus RHE, the NH_3_ yield and FE gradually increased due to the gradually accelerated reaction kinetics of the NO_3_RR and high *H utilization rate during hydrogenation steps. However, the further increase of cathodic potential lowered NH_3_ yield and FE due to the enhanced HER. Our catalyst also demonstrated excellent NH_3_ yield and FEs in 0.3 M NO_3_^−^ compared with 0.1  and 0.5 M. The poor activity under lower NO_3_^−^ concentration could be attributed to the following two factors: the insufficient nitrate flux on the electrode surface restricts reaction kinetics, while the weakened mass transfer effect exacerbates competition from HER (Figure S26). As the NO_3_^−^ concentration increases, the mass transfer process becomes significantly enhanced, which promotes the adsorption of more *H to participate in subsequent hydrogenation reaction. Such competitive adsorption inhibits the HER process, and the NH_3_ yield rate and FE gradually increase. However, when NO_3_^−^ concentration reaches 0.5 M, the FE exhibits an unexpected decline. This phenomenon is attributed to insufficient *H supply, resulting in substantial accumulation of NO_2_^−^ intermediates.

In addition, the cyclic stability tests were also conducted at −0.75 V versus RHE over 50 consecutive cycles, with each cycle lasting half an hour. After each cycle, the working electrode was rinsed with deionized water, air‐dried naturally and reused without replacement. Compared to the first cycle, the NH_3_ yield decreased by only 0.37 mg·h^−1^·cm^−2^ after 50 cycles (Figure 3f), and there was also no detectable Cu or Co signals were observed in post‐reaction electrolyte solutions (Figure S27), confirming the stable coordination of metallic species within the COF framework. Furthermore, the SEM and TEM after cycle test revealed unchanged morphology and preserved (001) lattice fringes with 0.33 nm (Figures S28 and S29), while XRD patterns retained maintained characteristic diffraction peaks at 3.58° (Figure S30). These results demonstrate the excellent structural robustness of TTA‐TPH‐CuCo, where the anchored metal centers resist agglomeration, leaching and framework collapse during electrochemical measurements.

The superior performance could be elaborated by the following reasons. Firstly, the COFs are functioned as promising support to anchoring Cu and Co single atoms with coordinatively unsaturated sites, efficiently avoiding the process of pyrolysis and the aggregation of Cu and Co atoms with inherent high surface free energy. The ordered and stable structure of COFs and the dispersed active sites can inhibit the aggregation of metal atoms, thus enhancing the stability of the catalyst. Secondly, COFs are a type of crystalline porous organic polymer where predesigned organic linkers are precisely integrated into two‐dimensional extended structures with periodic frameworks and well‐defined pores utilizing dynamic covalent chemistry. By virtue of regular porous structures, precisely tunable pore sizes, long‐range ordered crystalline topological networks, and atomically dispersed metal sites, COFs could provide a unique research perspective for facilitating rapid electron transport, optimizing the mass transfer kinetics of NO_3_^−^, revealing complex reaction mechanism.

Based on the excellent NO_3_RR performance of TTA‐TPH‐CuCo, a Zn‐NO_3_^−^ battery was assembled with TTA‐TPH‐CuCo as the cathode and zinc foil as the anode to simultaneously eliminate nitrate, produce ammonia and generate power (Figure S31). Interestingly, the battery could achieve a NH_3_ yield of 52.4 µmol·cm^−2^·h^−1^ and power density of 1.2 mW·cm^−2^, advancing their significant potential for practical applications.

To identify the N source in NH₃ products and exclude potential contamination, the isotopic labeling experiments were performed using ^15^NO_3_^−^ and ^14^NO_3_^−^ as nitrogen sources to trace the origin of nitrogen in the produced NH_3_ (Figure 3g). In the ^1^H NMR spectra, the doublet and triplet peaks were attributed to ^15^NH_4_^+^ and ^14^NH_4_^+^, with coupling constants of 71.8  and 51.7 Hz, respectively. The control experiments under open‐circuit potential (OCP), nitrate‐free electrolyte and bare carbon paper conditions all showed undetectable ammonia signals (Figure S32).

The above experiments indicate that the obtained NH_3_ is from the electrocatalytic nitrate reduction reaction, rather than other sources such as the catalyst or the experimental setup. The NH_3_ yield were further determined via ^1^H NMR, with the corresponding calibration curve presented in Figure S33. The results were consistent with the performance obtained by the indophenol blue method (Figure S33d) when using ^15^NH_4_^+^ or ^14^NH_4_^+^ as a reactant, demonstrating the reliability of the two methods.

**Figure 4:**
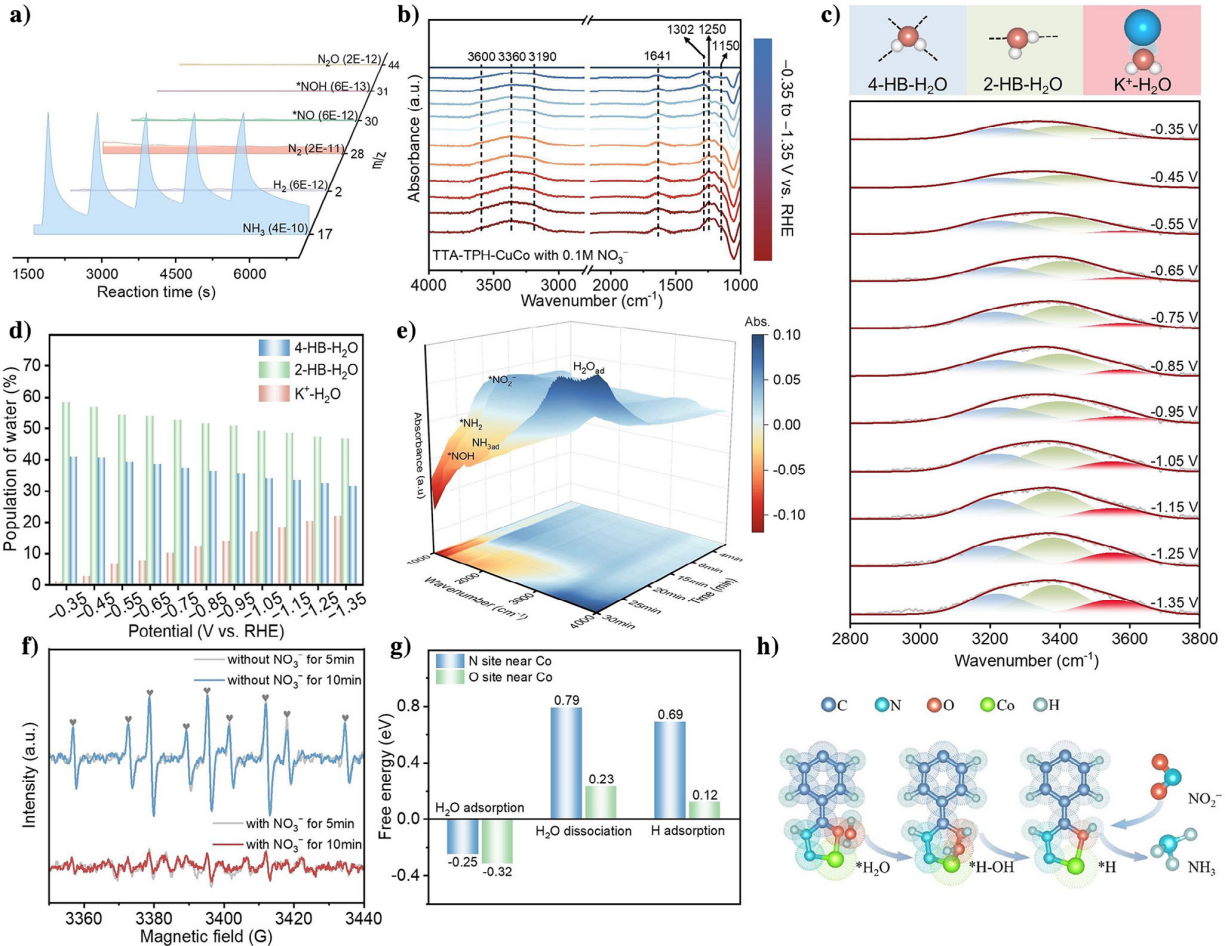
In situ characterizations and reaction mechanism. a) In situ DEMS patterns of TTA‐TPH‐CuCo. b) In situ ATR‐IRAS measurements under different potentials. c) Gaussian fitting on O‐H stretching bands of TTA‐TPH‐CuCo and the corresponding structures for 4‐HB‐H_2_O, 2‐HB‐H_2_O and K^+^‐H_2_O. d) The relative proportions of three types of water as a function of potential. e) In situ ATR‐IRAS measurements during 30‐min test (at −0.75 V vs. RHE). f) EPR spectra with and without 0.1 M NO_3_^−^. g) Energy barriers during the processes of H_2_O adsorption, dissociation and *H adsorption. h) The adsorption and dissociation process of water over the Co site.

In addition, to obtain information on intermediates and identify the reaction pathway of NO_3_RR over prepared catalysts, in situ differential electrochemical mass spectrometry (DEMS) was used to capture volatile gaseous molecules generated during the reaction. In the in situ DEMS measurements of TTA‐TPH‐CuCo (Figure 4a), the apparent signal of m/z 17 and weak peaks of m/z 2 were captured, which could be assigned to the main product NH_3_ and by‐product H_2_. Meanwhile, the NO_3_RR process over TTA‐TPH‐Cu exhibited notable HER activity, with substantial H_2_ evolution being detected in the DEMS measurement (Figures S34 and S35). In contrast, the TTA‐TPH‐Co catalyst demonstrated effective suppression of HER. Notably, the incorporation of Co sites in the bimetallic TTA‐TPH‐CuCo significantly enhanced the utilization efficiency of *H for the *NO hydrogenation, thereby effectively suppressing competitive HER. The DEMS also revealed characteristic signals of critical intermediates *NOH and *NH₂, with TTA‐TPH‐CuCo displaying enhanced signal intensities for both species (Figure S36). These findings conclusively verifying the tandem catalytic mechanism of TTA‐TPH‐CuCo in NO_3_RR. This mechanism achieves efficient cascade catalysis by efficiently matching the two‐step reaction, namely the two‐electron‐mediated intermediate conversion process and subsequent six‐electron hydrogenation stages, achieving optimal kinetic matching for enhanced catalytic efficiency. Additionally, critical reaction intermediates with a characteristic m/z value of 31 was also detected through in situ DEMS, corresponding to either *NOH or *NHO, which has great effect on the subsequent reaction.

In order to further confirm the reaction pathway, in situ attenuated total reflection‐surface enhanced infrared absorption spectroscopy (ATR‐IRAS) was used to confirm solution‐phase intermediates and adsorbed species on the electrode surface. The in situ ATR‐IRAS measurements were conducted in a 0.5 M K_2_SO_4_ solution containing 0.1 M KNO_3_. The catalytic behavior at different potential was systematically investigated over a cathodic potential window spanning from −0.35 to −1.35 V versus RHE (Figure 4b). The controlled‐potential electrolysis was maintained at −0.75 V versus RHE for 60‐min duration were also explored (Figures 4e and S37). The peaks at 1150,^[^ ^77^ ^]^ 1250,^[^ ^78^ ^]^ and 1302^[^ ^79^ ^]^ cm^−1^ are attributed to *NH_2_, *NO_2_, and *NOH, while the gradually increasing peak at 1641^[^ ^80^ ^]^ cm^−1^ indicates the accumulation of adsorbed NH_3_. In the broad overlapping absorption band in the range of 3000 to 3700 cm^−1^,^[^ ^81^ ^]^ the peaks at 3600, 3360, and 3190 cm^−1^ are attributed to v(N‐H), v(O‐H) of adsorbed H_2_O and NOH, respectively. With increasing applied potential or prolonged reaction time, the intensities of these key intermediates increased, clearly indicating the active progression of the NO_3_RR process from NO_3_^−^ to NH_3_. In combination the in situ DEMS with ATR‐IRAS results, we could conclude that the conversion of NO_3_^−^ to NH_3_ proceeds via the following possible pathways: *NO_3_ → *NO_2_ → *NO → *NOH →*N →*NH →*NH_2_ →*NH_3_.

In a neutral system, the hydrogen ions (*H) requisite for the hydrogenation process during the NO_3_RR predominantly stem from the interfacial water adsorbed on the catalyst surface. As shown in Figure 4c,d, the in situ ATR‐IRAS analysis of TTA‐TPH‐CuCo in the 3000 to 3700 cm^−1^ region revealed a characteristic vibrational signal assignable to interfacial water,^[^ ^82^, ^83^, ^84^, ^85^ ^]^ which was resolved through Gaussian deconvolution into three states: tetra‐coordinated hydrogen‐bonded water (4‐HB‐H_2_O), bi‐coordinated hydrogen‐bonded water (2‐HB‐H_2_O) and alkali metal‐ion‐coordinated water (K^+^‐H_2_O).^[^ ^79^, ^86^ ^]^ This multimodal water coordination architecture suggests the formation of dynamic hydration layers at the electrocatalyst‐electrolyte interface, where the K^+^‐H_2_O is closely associated with water dissociation.^[^ ^87^, ^88^, ^89^ ^]^ Notably, the area fraction of K^+^‐H_2_O consistently maintained positive increase among interfacial hydration components, exhibiting a remarkable enhancement from 0.9 % to 21.9 % when decreasing the applied potential. This result suggests that TTA‐TPH‐CuCo facilitates the dissociation of *H_2_O, generating a sufficient *H supply on the catalyst surface, thereby promoting the efficient progression of NO_3_RR.

The sufficient supply of *H is the key to ensure the steady and efficient progress of the NO_3_RR. We then further monitor the *H signal during NO_3_RR process via electron paramagnetic resonance (EPR) spectroscopy by using 5,5‐dimethyl‐1‐pyrroline‐N‐oxide (DMPO) as *H trapping agent. In a 0.5 M K_2_SO_4_ solution, nine characteristic peaks corresponding to *H with an intensity ratio approaching 1:1:2:1:2:1:2:1:1 were achieved. As evidenced by Figure S38, introduction of NO_3_^−^ triggers significant attenuation of *H signals in the TTA‐TPH‐CuCo system, demonstrating active participation of these *H during the hydrogenation of NO_3_RR intermediates. This substantial *H consumption correlates with enhanced catalytic activity and accelerated reaction dynamics observed in the bimetallic CuCo catalyst system. In contrast, both the supply and consumption levels of *H in the TTA‐TPH‐Cu were inferior to those in the Co‐containing system, which hinder the hydrogenation of the intermediates, resulting in lower activity (with a FE of 64.5% in 0.1 M NO_3_^−^ and 0.5 M K_2_SO_4_). Furthermore, the sustained and stable supply of *H is critical for enhancing activity and efficiency. To monitor the temporal evolution of *H content, EPR spectra were recorded at 5 and 10 min, represented by gray and colored lines, respectively (Figure 4f). The content of *H in the TTA‐TPH‐CuCo system remained nearly constant after 10 min, indicating its ability to stably and continuously supply *H, which supports the robust and efficient NO_3_RR performance of TTA‐TPH‐CuCo.

In addition, the theoretical calculation of the H_2_O dissociation pathway further confirm that Co and its adjacent N and O atoms play important roles in the generation and utilization of *H. As shown in Figure 4g, the H_2_O adsorption and dissociation on the N and O atoms adjacent to the Co site are much more favorable than those on the Cu site (Figure S39), and the H_2_O adsorption and dissociation on the O atom are much more energetically favorable than N atom. As shown in Figure 4h, H_2_O is adsorbed on the O atom beside Co and dissociates into *H‐OH. The generated *H is also more stably adsorbed on the O atom, forming an environment rich in *H. This result demonstrated that the ‐N‐Co‐O‐ catalytic interface could enable efficient *H supply for the NO_3_RR reaction, then the generated *H can be immediately utilized in the hydrogenation process and maintain a dynamic equilibrium of *H concentration.

**Figure 5:**
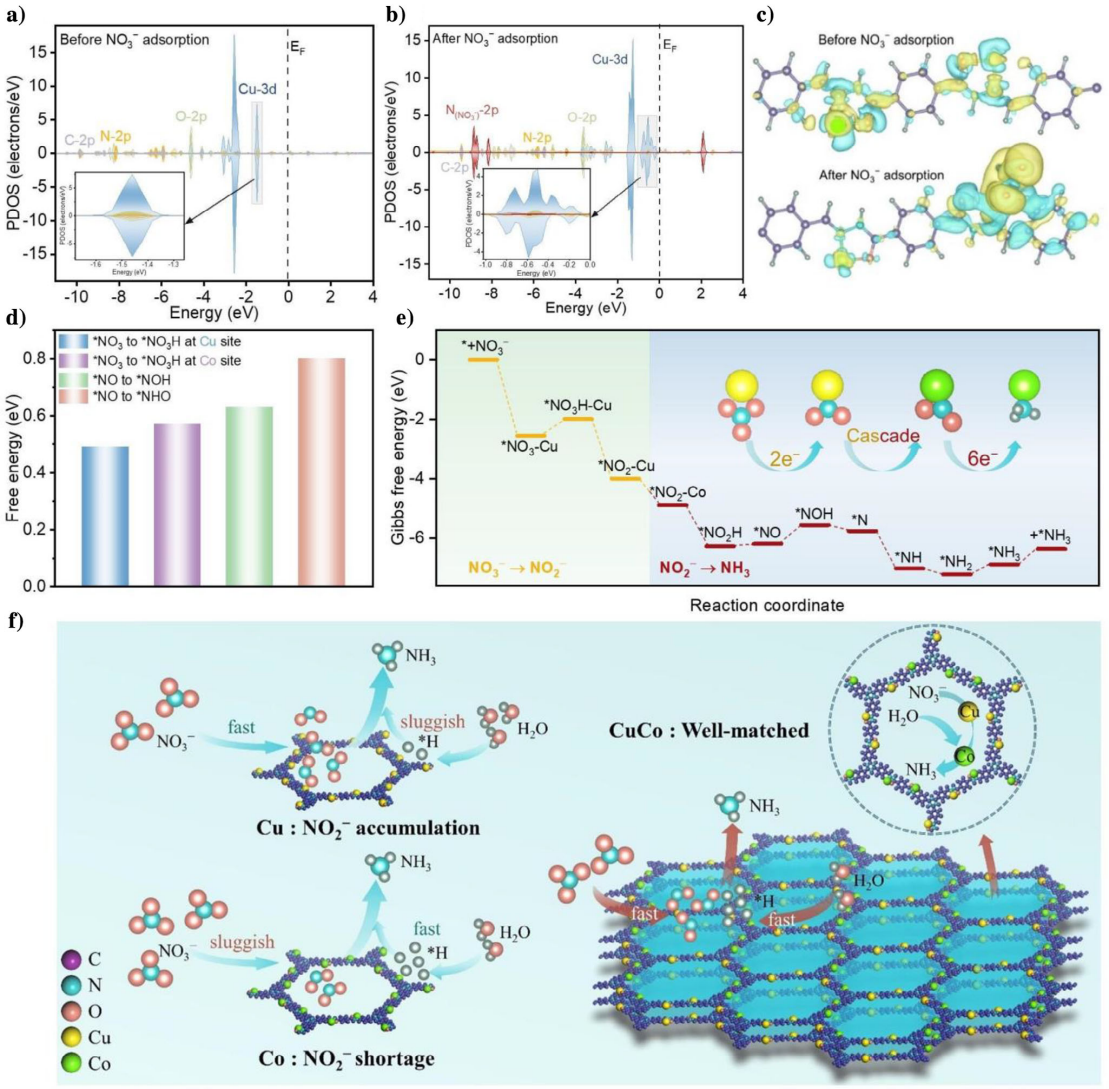
Theoretical calculations and reaction pathway. PDOS spectra of TTA‐TPH‐CuCo (a) before and (b) after NO_3_^−^ adsorption. c) Differential charge density of TTA‐TPH‐CuCo before and after NO_3_^−^ adsorption. d) Energy barriers for the formation of *NO_3_H and *NHO/*NOH intermediate over TTA‐TPH‐CuCo active sites. e) Free energies of the NO_3_RR reaction pathway at Cu site (NO_3_^−^ to NO_2_^−^) and Co site (NO_2_^−^ to NH_3_). f) The schematic illustration of the tandem mechanism on TTA‐TPH‐CuCo.

We further investigated the interaction between NO_3_^−^ and TTA‐TPH‐CuCo catalyst through theoretical calculations. Firstly, we utilized the projected density of states (PDOS) to confirm the local electronic states near the Fermi level (E_F_) of TTA‐TPH‐ CuCo before and after the adsorption of NO_3_^−^. The orbital overlap of Cu‐3d, N‐2p and O‐2p near the E_F_ indicates the formation of new hybrid orbitals through strong electronic interactions, which ensures the effective charge transfer of the catalyst (Figure 5a). After NO_3_^−^ adsorption, the N (NO_3_^−^)‐2p orbitals of NO_3_^−^ overlap with the above orbitals, revealing significant electronic coupling between NO_3_^−^ and Cu active centers in the TTA‐TPH‐CuCo catalyst (Figure 5b). The upshifted Cu‐3d orbitals after NO_3_^−^ adsorption further indicated the strong interaction between NO_3_^−^ and Cu catalytic sites, which holds substantial significance for the subsequent NO_3_RR reaction. To elucidate the interfacial charge transfer, the differential charge density of TTA‐TPH‐CuCo before and after NO_3_^−^ adsorption was calculated in Figure 5c, where cyan represents the loss of charge and yellow represents the gain of charge. It could be seen that there was an obvious electron transfer between the metallic Cu site and NO_3_^−^. The bader calculation shown that the electron of Cu is decreased from 10.41 to 10.22, featuring an electron accumulation on NO_3_^−^ (Δq = 0.19 e^−^).

Furthermore, theoretical calculations in Figure 5d revealed that the *NO→*NOH step had a lower endothermic energy barrier (0.63 eV) compared to the *NO → *NHO step (0.8 eV), indicating that the former is a more favorable pathway and agreeing well with the in situ experiments results. For the rate‐determining *NO_3_ → *NO_3_H hydrogenation step, the Cu site exhibits a lower activation barrier (ΔG = 0.49 eV) compared to the Co site (ΔG  = 0.57 eV). This thermodynamic preference, combined with enhanced NO_3_^−^ adsorption due to favorable orbital hybridization, confirming that Cu center is the primary center for initiating the two‐electron reduction pathway. As shown in Figure 5e, the theoretical calculations further revealed distinct catalytic function of Cu and Co sites in governing the NO_3_RR pathway energetics. Critical to subsequent steps, the adsorption of *NO_2_ intermediate shows evident site dependence, with Co exhibiting stronger adsorption energy (−4.89 eV) than Cu (−4.00 eV). The free energy change of the process from Cu site to Co site is −0.89 eV, which is a spontaneous process and thermodynamically favorable. For the *NO_2_ → *NO_2_H hydrogenation step, the Co site exhibits a beneficial energy barrier (ΔG = −1.38 eV) compared to the Cu site (ΔG = −1.23 eV). This differential binding enables tandem reaction: initial nitrate activation and NO_3_^−^→ NO_2_^−^ reduction occur at Cu sites, while the Co domain preferentially drives the subsequent six electron transfer steps. Therefore, the adsorbed NO_2_^−^ intermediates migrate to Co sites for sequential hydrogenation via the energetically favorable *NOH pathway (ΔG = 0.63 eV), ultimately completing the NO_2_^−^ to NH_3_ process.

Differential charge density analysis also reveals the electron transfer mechanism at active centers (Figure S40). The NO_3_^−^ adsorbed on Cu sites (electron transfer from Cu→NO_3_^−^) initiates reduction, forming *NO_2_intermediates. These intermediates preferentially adsorb on Co sites, enabling Co as electron donors to drive *NO_2_ reduction. In this process, the tandem Cu and Co active sites, as the key mediators of electron transfer, ultimately enabling efficient conversion from NO_3_^−^ to NH_3_. These simulation results are in excellent agreement with our experimental conclusions from in situ DEMS and in situ ATR‐IRAS, clearly clarifying the tandem catalytic mechanism of TTA‐TPH‐CuCo in NO_3_RR (Figure 5f). Firstly, the mismatch rates between the rapid conversion process of NO_3_^−^ on TTA‐TPH‐Cu and the subsequent sluggish hydrogenation process leads to the accumulation of NO_2_^−^. The shortage of NO_2_^−^ on TTA‐TPH‐Co also prevents the realization of high‐efficient NH_3_ production performance. For TTA‐TPH‐CuCo, the Cu site significantly adsorbs NO_3_^−^, serving as the adsorption and activation center in the NO_3_RR process. Subsequently, the ‐N‐Co‐O‐ interface simultaneously promotes the dissociation of H₂O and the supply of *H to ensure the steady progress of the subsequent hydrogenation process. The tandem catalysis of Cu and Co optimizes the intermediate steps and transition states of the reaction, thereby accelerating the kinetics of the proton‐coupled electron transfer reaction.

## Conclusion

In summary, we have designed and fabricated a bimetallic TTA‐TPH‐CuCo catalyst with tandem active sites for the cascade conversion of nitrate to ammonia. Compared with the monometallic counterparts, TTA‐TPH‐CuCo achieves a high NH_3_ yield rate of 20.8 mg·h^−1^·cm^−2^ with FE of 92.16% in 0.3 M nitrate, outperforming most of the reported NO_3_RR electrocatalysts in the literature. Through in situ DEMS, in situ ATR‐IRAS and theoretical calculations, we have elucidated the cascade mechanism occurring over the TTA‐TPH‐CuCo. The Cu sites function as the activation centers for adsorption and the initial 2e^−^ process. The ‐N‐Co‐O‐ interface promotes water adsorption and dissociation, facilitates the formation of *H‐rich interface, and boosts the subsequent 6e^−^ process, thus accelerating efficient synthesis of ammonia. This work not only emphasizes the achievement of efficient NO_3_RR through matching a two‐step tandem reaction, but also paves the way for designing highly efficient electrocatalysts for NO_3_RR and possible other reactions in which high selectivity is desired.

## Supporting Information

The detailed experimental section, additional figures tables are listed in the Supporting Information file. The authors have cited additional references within the Supporting Information.

## Conflict of Interests

The authors declare no conflict of interest.

## Supporting information



Supporting Information

## Data Availability

The data that support the findings of this study are available from the corresponding author upon reasonable request.
