# Supplementary material for: Cascade Electrocatalytic Reduction of Nitrate to Ammonia Using Bimetallic Covalent Organic Frameworks with Tandem Active Sites
Source: Angew Chem Int Ed Engl. 2025 Jun 18;64(32):e202507956. doi: 10.1002/anie.202507956 (PMC12322651; doi:10.1002/anie.202507956)
Supplement: Supplementary file 1 — Supporting Information [file ANIE-64-e202507956-s001.docx]

***Supporting Information***

**Cascade Electrocatalytic Reduction of Nitrate to Ammonia using Bimetallic Covalent Organic Frameworks with Tandem Active Sites**

Jian Zhong,^[a]^ Haiyan Duan,*^[a]^ Mingquan Cai,^[a]^ Ying Zhu,^[a]^ Zhenlin Wang,^[a]^ Xingchi Li,^[a]^ Zhengliang Zhang,^[a]^ Wenqiang Qu,^[a,c]^ Kai Zhang,^[a]^ Donglin Han,^[a]^ Danhong Cheng,^[a]^ Yongjie Shen,^[d]^ Ming Xie,^[e]^ Emiliano Cortes*,^[b]^ Dengsong Zhang*^[a]^

[a] J. Zhong, Assoc.Prof. H. Duan, M. Cai, Y. Zhu, Z. Wang, X. Li, Z. Zhang, W. Qu, K. Zhang, D. Han, Prof. D. Cheng, Prof. D. Zhang
International Joint Laboratory of Catalytic Chemistry, State Key Laboratory of Advanced Special Steel, Innovation Institute of Carbon Neutrality, Department of Chemistry, College of Sciences, Shanghai University, Shanghai 200444, People’s Republic of China
E-mail: [haiyanduan@shu.edu.cn](mailto:haiyanduan@shu.edu.cn) and [dszhang@shu.edu.cn](mailto:dszhang@shu.edu.cn)

[b] Prof. E. Cortés
Nanoinstitute Munich, Faculty of Physics, Ludwig-Maximilians-Universität (LMU), Munich 80539, Germany.
E-mail: [Emiliano.Cortes@lmu.de](mailto:Emiliano.Cortes@lmu.de)

[c] Dr. W. Qu
Department of Chemistry, University of Toronto, 80 St. George Street, Toronto, ON M5S 3H6, Canada

[d] Dr. Y. Shen
Institute for Chemical Reaction Design and Discovery (WPI-ICReDD), Hokkaido University, Sapporo 001-0021, Japan

[e] Dr. M. Xie
Department of Chemical Engineering, University of Bath, Bath BA2 7AY, U.K.

**Contents**

1. Materials and Reagents
2. Experimental Section
3. Figures and Tables

Figure S1. AFM images of TTA-TPH and the corresponding height curves.

Figure S2. TEM images of TTA-TPH and TTA-TPH-CuCo.

Figure S3. HR-TEM images of TTA-TPH-CuCo.

Figure S4. SEM images and the elemental mapping images of all samples.

Figure S5. FT-IR spectra of all samples.

Figure S6. ^13^C solid-state NMR spectrum of TTA-TPH.

Figure S7. The survey XPS spectra of all samples.

Figure S8. XPS spectra of the N 1s spectra for all samples.

Figure S9. XPS spectra of Cu 2p for TTA-TPH-Cu and Co 2p for TTA-TPH-Co.

Figure S10. WT-EXAFS plots of Cu for Cu foil and Cu_2_O.

Figure S11. The EXAFS R-space fitting curves of Cu for various samples.

Figure S12. The EXAFS k-space fitting curves of Cu for various samples.

Figure S13. WT-EXAFS plots of Co for Co foil and Co_2_O_3_.

Figure S14. The EXAFS R-space fitting curves of Co for various samples.

Figure S15. The EXAFS k-space fitting curves of Co for various samples.

Figure S16. LSV curves in 0.5 M K_2_SO_4_ with 0.1 M NO_3_^−^.

Figure S17. CV curves of various samples at varying scan rates within the non-Faradaic region.

Figure S18. The turnover frequency of TTA-TPH-CuCo, TTA-TPH-Cu and TTA-TPH-Co.

Figure S19. The actual mass proportions of CuCo under different addition ratios.

Figure S20. Determination of NH_3_.

Figure S21. NH_3_-FE at different CuCo proportions.

Figure S22. Determination of nitrite (NO_2_^−^).

Figure S23. Determination of hydroxylamine (NH_2_OH).

Figure S24. FE of NH_3_, NO_2_^−^ and H_2_ over TTA-TPH-CuCo, TTA-TPH-Cu and TTA-TPH-Co.

Figure S25. The pH curve of TTA-TPH-CuCo during the 30 minutes electrochemical test.

Figure S26. The LSV curves of TTA-TPH-CuCo with different concentrations of NO_3_^−^.

Figure S27. The standard ICP curves of Cu ions and Co ions.

Figure S28. SEM image and the elemental mapping images after the NO_3_RR process.

Figure S29. TEM and HR-TEM images of TTA-TPH-CuCo after the electrochemical test.

Figure S30. PXRD pattern of TTA-TPH-CuCo before and after NO_3_RR process.

Figure S31. Zn-NO_3_^−^ battery.

Figure S32. NO_3_RR performance over TTA-TPH-CuCo and CP under different conditions.

Figure S33. The ^1^H NMR signals and calibration curves of standard NH_4_^+^ solutions, and

comparison of NH_3_ yield and FE using UV-Vis and NMR methods.

Figure S34. *In situ* DEMS patterns of TTA-TPH-Cu and TTA-TPH-Co.

Figure S35. *In situ* DEMS of H_2_ signal for various samples.

Figure S36. *In situ* DEMS of *NOH and *NH_2_ signal for various samples.

Figure S37. *In situ* ATR-IRAS measurements during 60 minutes test.

Figure S38. EPR spectra with and without 0.1 M NO_3_^−^.

Figure S39. Energy barriers during the processes of H_2_O adsorption.

Figure S40. The electrons transfer process during the NO_3_RR process.

Table S1. Atomic coordinates of the AA-stacking mode of TTA-TPH COF.

Table S2. EXAFS fitting parameters at the Cu K-edge for various samples.

Table S3. EXAFS fitting parameters at the Co K-edge for various samples.

Table S4. Comparison of electrocatalytic NO_3_RR performance over the recently reported

electrocatalysts.

1. Materials and Reagents

**Chemical reagents.** All chemical reagents were used as received, without any further purification.

2. Experimental Section

**Synthesis of TTA-TPH.** 4,4’,4’’- (1,3,5-triazine-2,4,6-triyl) tribenzaldehyde (19.7 mg, 0.05 mmol), 1,3,5-trimethylbenzene (9.7 mg, 0.05 mmol), mesitylene (0.3 mL), 1,4-dioxane (0.7 mL) and 6 M trifluoroacetic acid (0.1 mL) were added in a pyrex tube. After degassed through three freeze-pump-thaw cycles, the pyrex tube was heated at 120 °C for 72 hours. The precipitate was collected by filtration and washed with DMF, and ethanol for several times. Subsequently, it was dried under vacuum at 80 °C overnight to yield the yellow TTA-TPH.

**Synthesis of TTA-TPH-Cu and TTA-TPH-Co.** TTA-TPH (20 mg), ethanol (60 mL), Cu(OAc)_2_·H_2_O (59.9 mg) or Co(OAc)_2_·4H_2_O (74.7 mg), were added to a three-necked flask. Using nitrogen to eliminate air, the mixture was stirred and heated at 60 °C for 12 hours. After cooling to room temperature, the solid was collected by filtration in the same manner. The solid was washed sequentially with water and ethanol, and then dried under vacuum at 80 °C overnight to obtain the sample.

**Synthesis of TTA-TPH-Cu_x_Co_y_.** Cu(OAc)_2_·H_2_O (15.0 mg) and Co(OAc)_2_·4H_2_O (56.0 mg) with different ratios was added to obtain bimetallic catalyst with all other steps unchanged.

**Materials characterizations.** The morphology of the materials was examined using a field emission scanning electron microscope (SEM, Sigma-300) and a transmission electron microscope (TEM, JEOL JEM-2100), with energy dispersive spectroscopy (EDS) mapping images collected for elemental analysis. Atomic force microscopy (AFM, Park system AFM-NX10) was used to analyze the surface morphology and roughness of the materials. Powder X-ray diffraction (PXRD) data were collected at room temperature using a Rigaku D/Max-RB diffractometer with Cu-Kα radiation ( λ = 1.5418 Å, 40 kV, 40 mA) over a scanning range of 2° to 40°, with a scan speed of 1° per minute and a step size of 0.02°. X-ray photoelectron spectroscopy (XPS) measurements were performed using a PHI-5300 instrument. ^1^H nuclear magnetic resonance (NMR) spectra were obtained using a Bruker 600 MHz nuclear magnetic resonance spectrometer. Solid-state NMR ^13^C spectroscopy was conducted on a Bruker Avance Neo 400WB. Inductively coupled plasma optical emission spectrometer (ICP-OES) analysis of the elemental content in the samples was performed using an Agilent 720ES. N_2_ adsorption/desorption measurements were carried out on a Quadrasorb-evo apparatus. The obtained data were processed using the Brunauer-Emmett-Teller (BET) equation and the Non-local Density Functional Theory (NLDFT) model. Ultraviolet-visible spectrophotometry (UV-vis, Agilent, Cary5000) was used to determine the ammonia content. The hydrogen gas generated in the reaction was quantitatively analyzed using a gas chromatograph (Agilent 8890). Differential electrochemical mass spectrometry (DEMS, QAS 100) and in situ attenuated total reflectance infrared spectroscopy (ATR-IRAS, Bruker INVENIO R) were employed to detect intermediates and analyze the reaction pathway.

**Electrochemical measurements.**

**Working electrode preparation.** A mixture of catalyst (2 mg) and carbon black (1 mg) was ground and then added to 220 µL of ethanol, 160 µL of water, and 20 µL of Nafion perfluorinated resin (5 wt.% in mixture alcohols and water) to prepare the catalyst ink for the working electrode. Subsequently, 50 µL of the catalyst ink was drop-coated onto carbon paper, covering an area of 1×1 cm².

**Electrochemical nitrate reduction reaction.** All electrochemical experiments were conducted at room temperature using an electrochemical workstation (CHI 760E) and a two-chamber H-cell. In the H-cell tests, a proton exchange membrane (Nafion 117) was employed to effectively facilitate proton transport while preventing the permeation of gases and liquids. The electrolytes used in both the cathode and anode chambers comprised 30 mL of 0.5 M K_2_SO_4_ mixed with varying concentrations of KNO_3_. A saturated calomel electrode (SCE) served as the reference electrode in the cathode chamber, while the working electrode consisted of carbon paper coated with catalyst. A platinum sheet electrode was utilized as the counter electrode in the anode chamber. The nitrate reduction reaction was conducted using the potentiostatic method at different potentials for 30 minutes (with 80% IR compensation). Moreover, only the potentiostatic test included IR compensation, while all other electrochemical tests were conducted without IR compensation.

**Evaluation of electrochemical active surface area (ECSA).** To evaluate the intrinsic catalytic activity of the catalysts, cyclic voltammetry (CV) curves were recorded at different scan rates, and the double-layer capacitance (C*_dl_*_)_ was determined through linear fitting. Since the specific capacitance (C*_s_*_)_ of the catalyst surface exhibits negligible variation, C*_dl_* is directly proportional to the ECSA.

The charging current (I_c_) at different scan rates (V) is related to C_dl_ as follows:

Ic=C*_dl_*⋅V

Here, C_s_ represents the specific capacitance of the sample under identical conditions. The relationship between ECSA, C_dl_ and C_s_ is expressed as:

ECSA = $\frac{c_{dl}}{c_{s}}$

**In situ electrocatalytic measurements.**

**DEMS.** Using the same method as for preparing the working electrode, the ink was prepared and drop-coated onto the surface of a gold-sputtered PTFE current collector, which was then dried and used as the working electrode. A platinum wire electrode served as the counter electrode, and SCE was used as the reference electrode. The tests were conducted in 0.1 M KNO_3_ at −0.75 V vs. RHE for 150 seconds, during which mass signals were collected and analyzed. After the test, the system was allowed to stabilize before performing another test under the same conditions. This procedure was repeated five times in total.

**ATR-SEIRAS.** A layer of gold nanoparticles exhibiting surface enhancement effects was deposited on single-crystal silicon to serve as a conductive substrate. The catalyst was formulated as an ink and drop-coated onto the substrate, acting as the working electrode. A platinum wire electrode served as the counter electrode, while SCE was utilized as the reference electrode. Signals were collected in two modes: across various potentials (−0.35 to −1.35 V vs. RHE) and at a constant potential of −0.75 V vs. RHE, with continuous reactions lasting 60 minutes and 64 scans recorded every minute. The information on the adsorption states and intermediate products of the electrocatalyst was obtained.

**Product analysis.**

**Quantification of ammonia.** The concentration of NH_3_ was determined using the indophenol blue method. Typically, a 1 mL aliquot of the cathodic electrolyte was diluted to a specific volume. Subsequently, 2 mL of the diluted solution was mixed with 2 mL of reagent A (containing sodium hydroxide, sodium citrate, and salicylic acid), 1 mL of reagent B (containing sodium hypochlorite solution), and 0.2 mL of reagent C (containing sodium nitroferricyanide dihydrate). After allowing the color reaction to proceed at room temperature for 1 hour, the resulting solution was analyzed using a UV-visible spectrophotometer, measuring the absorbance at 655 nm to determine the concentration of ammonia.

In order to accurately measure the concentration of NH_4_^+^ generated after the NO_3_RR process, a 600 MHz proton nuclear magnetic resonance spectrometer (^1^H NMR) was used for the test, and the results were compared with those obtained by the indophenol blue method. After acidifying the known standard solution, it was mixed with an aqueous solution of maleic acid, an aqueous solution of sulfuric acid, and deuterated dimethyl sulfoxide (DMSO-d_6_), and then analyzed by ^1^H NMR measurement. The integral ratio of the peak area of the ammonium ion product to that of maleic acid was analyzed to establish a standard curve. The cathode solution to be tested was treated using the same method as described above, and the content of ammonia was quantitatively determined.

**Calculation of the yield rate and the Faradaic efficiency of NH_3_.**

The NH_3_ yield rate:

r(NH_3_) = $\frac{c_{{NH}_{3}}\times V}{t \times s}$

The Faradaic efficiency of NH_3_:

FE (%) = $\frac{8 \times F \times c_{{NH}_{3}}\times V}{Q_{total}}$

Where c represents the concentration of ammonia in the electrolyte after the reaction, V is the volume of the electrolyte (30 mL), t is the time of testing (0.5 h), S is the actual effective area of the working electrode (1 cm^2^), F is Faraday constant (96485 C mol^−1^), *Q_total_* is the integral of i-t curve.

**Determination of Hydroxylamine (NH_2_OH).** Initially, add 1.0 mL of PBS buffer (pH = 7.4) and 1% 8-hydroxyquinoline respectively to 1 mL of standard NH_2_OH solutions with different concentrations. After thorough mixing by vigorous shaking, add 1.0 mL of 0.1 M K_2_CO_3_. Then heat the mixture at 100 °C for 1 minute, and let it stand for 10 minutes after taking it out. Take the catholyte after the reaction, dilute it, and treat it using the same method as above. If NH_2_OH is present, the solution will change from light yellow to blue-green, showing an absorption peak at ~705 nm. Plot the peak absorbance against the NH_2_OH concentration to obtain a calibration curve, and calculate the Faradaic efficiency of NH₂OH using the following equations:

FE (%) = $\frac{n \times F \times c_{{NH}_{2}OH}\times V}{Q_{total}}$

**Determination of Nitrite (NO_2_^−^).** An ion chromatograph (SoptopIC1820) furnished with an anion column (Shanghai Shunyuhengping Company) was utilized to detect nitrite, which served as by-products. The mobile phase consisted of a 3.6 mM aqueous solution of K_2_CO_3_, flowing at a rate of 0.6 mL·min^−1^. A series of standard nitrite solutions with different concentrations were prepared, and a calibration curve was obtained through fitting. 1 mL of the catholyte to be tested was taken and diluted 10 times. By comparing with the standard curve, the concentration of the solution to be tested was determined. The Faradaic efficiency of nitrite can be calculated as follows:

FE (%) = $\frac{n \times F \times c_{{NO}_{2}-}\times V}{Q_{total}}$

**Electron Paramagnetic Resonance measurement.** The electron paramagnetic resonance (EPR) tests in this study were carried out on the EPR200-PLUS model equipment (produced by China Guoyi Quantum). The test conditions are as follows: the test temperature was 298 K, the modulation frequency was 100 kHZ, the microwave power was 1 mW, the microwave frequency was 9.52 GHz, the scanning center was at 3398 G, the modulation amplitude was 1 G and the scanning width was 100 G.

**^15^N isotope labelling experiment.** In order to determine the nitrogen source in the NO_3_RR process, an isotope labeling experiment was carried out in this paper on a 600 MHz proton nuclear magnetic resonance spectrometer (^1^H NMR). Electrochemical reduction was conducted for 1 hour in a 0.5 mol·L^−1^ K_2_SO_4_ solution containing K^14^NO3 or K^15^NO3, and the catholyte after the reaction was collected for later use. After acidifying the collected catholyte, maleic acid and DMSO-d_6_ were added, and then a ^1^H NMR test was performed.

**Assembly of Zn-NO_3_**^−^ **battery.** The Zn-NO_3_^−^ battery was constructed in an H-cell with the TTA-TPH-CuCo electrocatalyst serving as the cathode and a Zn foil as the anode. The cathode electrolyte was a 0.5 M K_2_SO_4_ solution containing 0.1 M KNO_3_, and the anode electrolyte was 0.1 M KOH. The performance of the battery was evaluated through a series of electrochemical tests, such as the Open Circuit Potential Test (OCPT), Linear Sweep Voltammetry (LSV), Multi-current tests, and so on.

**DFT calculations.** We used the DFT as implemented in the Vienna Ab initio simulation package (VASP) in all calculations. The exchange-correlation potential is described by using the generalized gradient approximation of Perdew-Burke-Ernzerhof (GGA-PBE). The projector augmented-wave (PAW) method is employed to treat interactions between ion cores and valence electrons. The plane-wave cutoff energy was fixed to 450 eV. Given structural models were relaxed until the Hellmann-Feynman forces smaller than −0.02 eV·Å^−1^ and the change in energy smaller than 10^−5^ eV was attained. Grimme’s DFT-D3 methodology was used to describe the dispersion interactions among all the atoms in adsorption models. The Gamma-centered k-points samplings were set to 1 × 1 × 1 for model. The vacuum space along the z-direction was set to be 12 Å

The Gibbs free energy change is defined as:

ΔG = ΔE + ΔZPE – TΔS

where ΔE is the electronic energy calculated with VASP, ΔZPE and ΔS are the zero-point energy difference and the entropy change between the products and reactants, respectively, and T is the temperature (298.15 K).

3. Figures and Tables


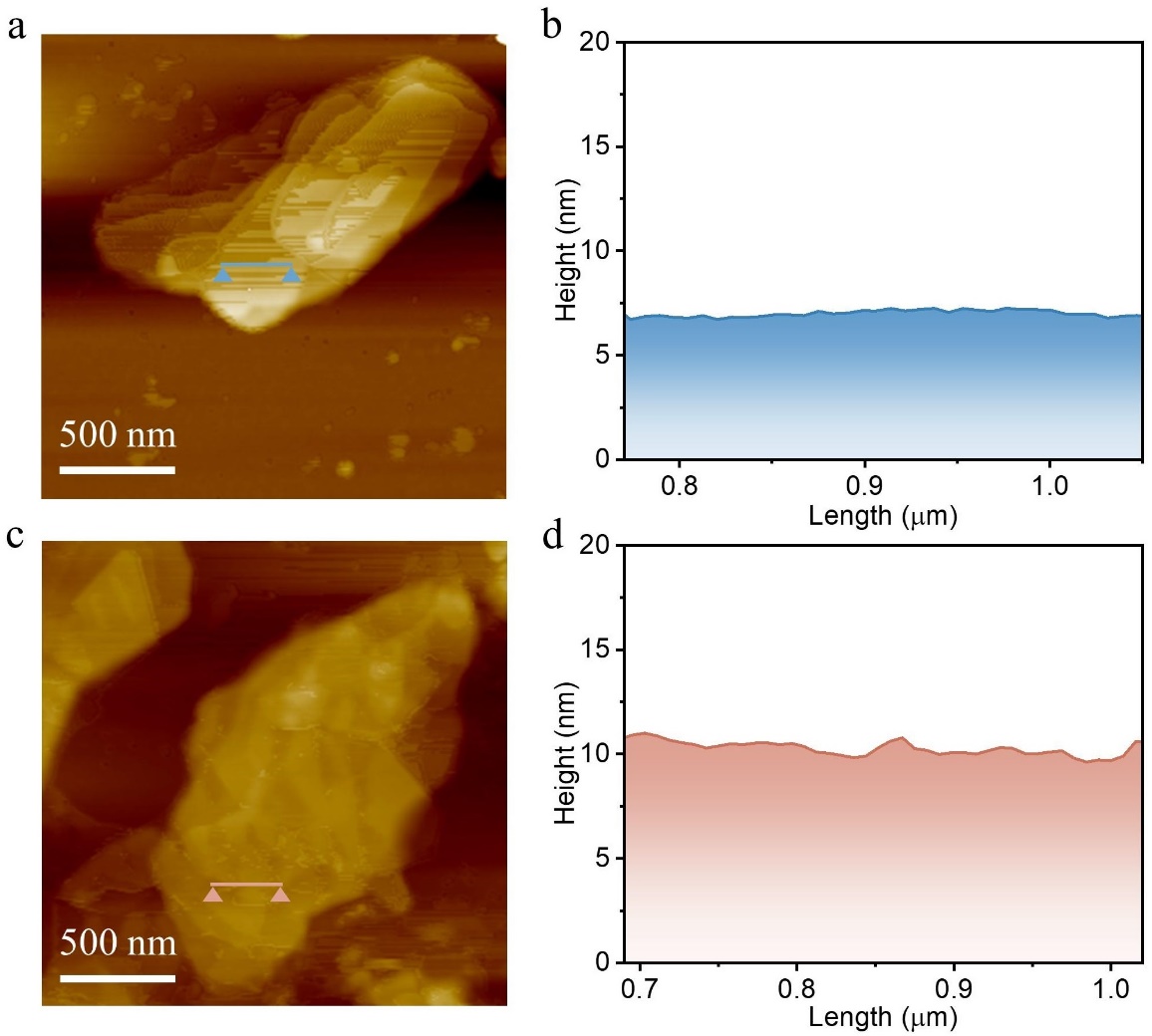


Figure S1. (a, c) AFM images of TTA-TPH and (b, d) the corresponding height curves relevant to the chosen areas.


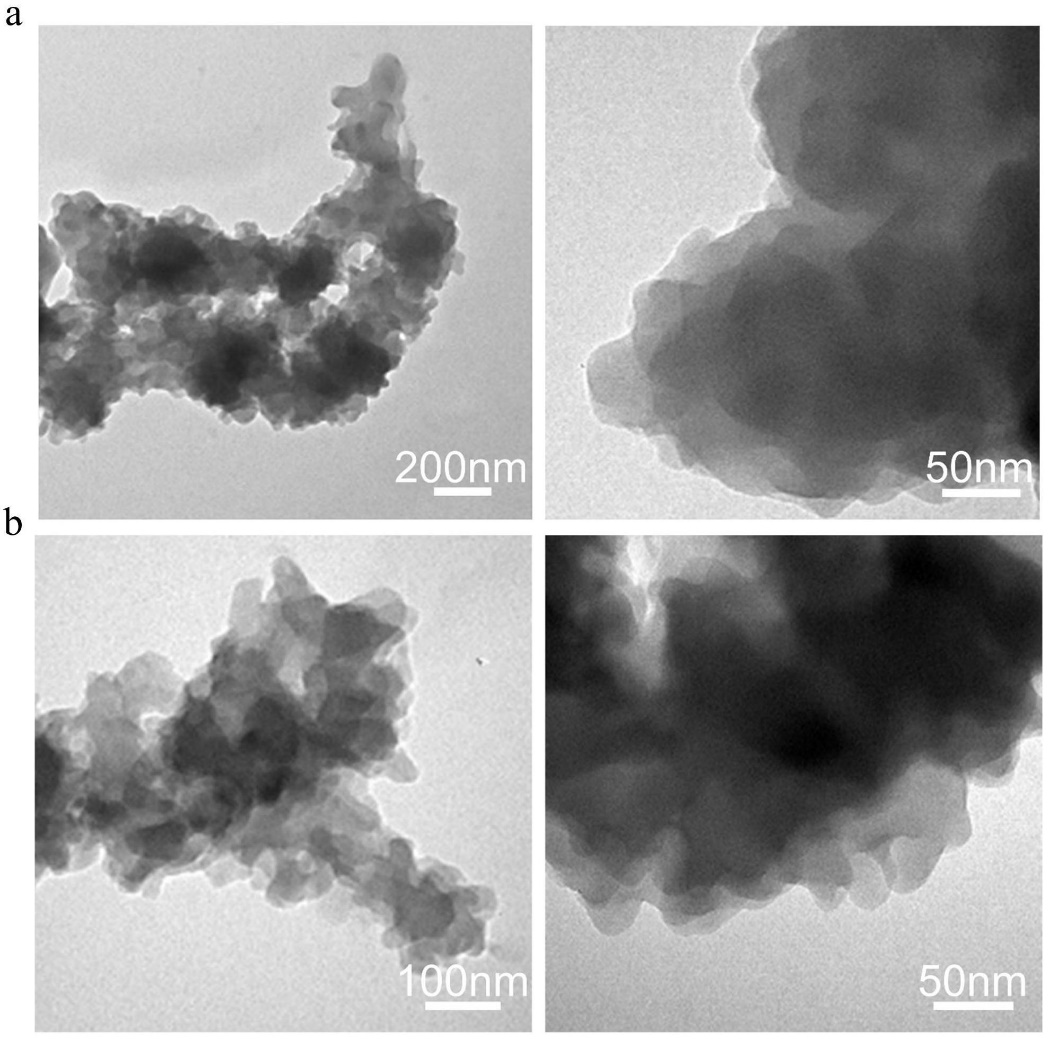


Figure S2. TEM images of (a) TTA-TPH and (b) TTA-TPH-CuCo.


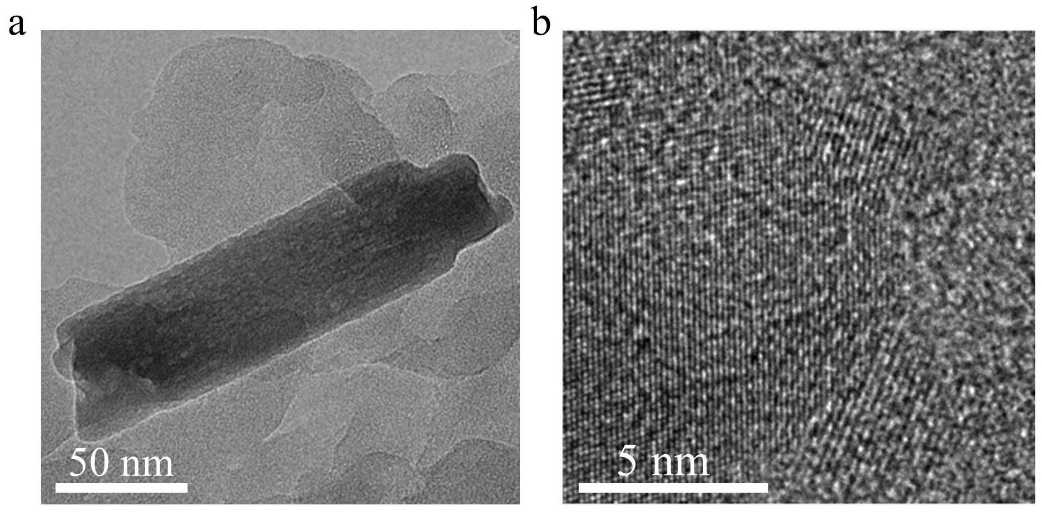


Figure S3. HR-TEM images of TTA-TPH-CuCo.


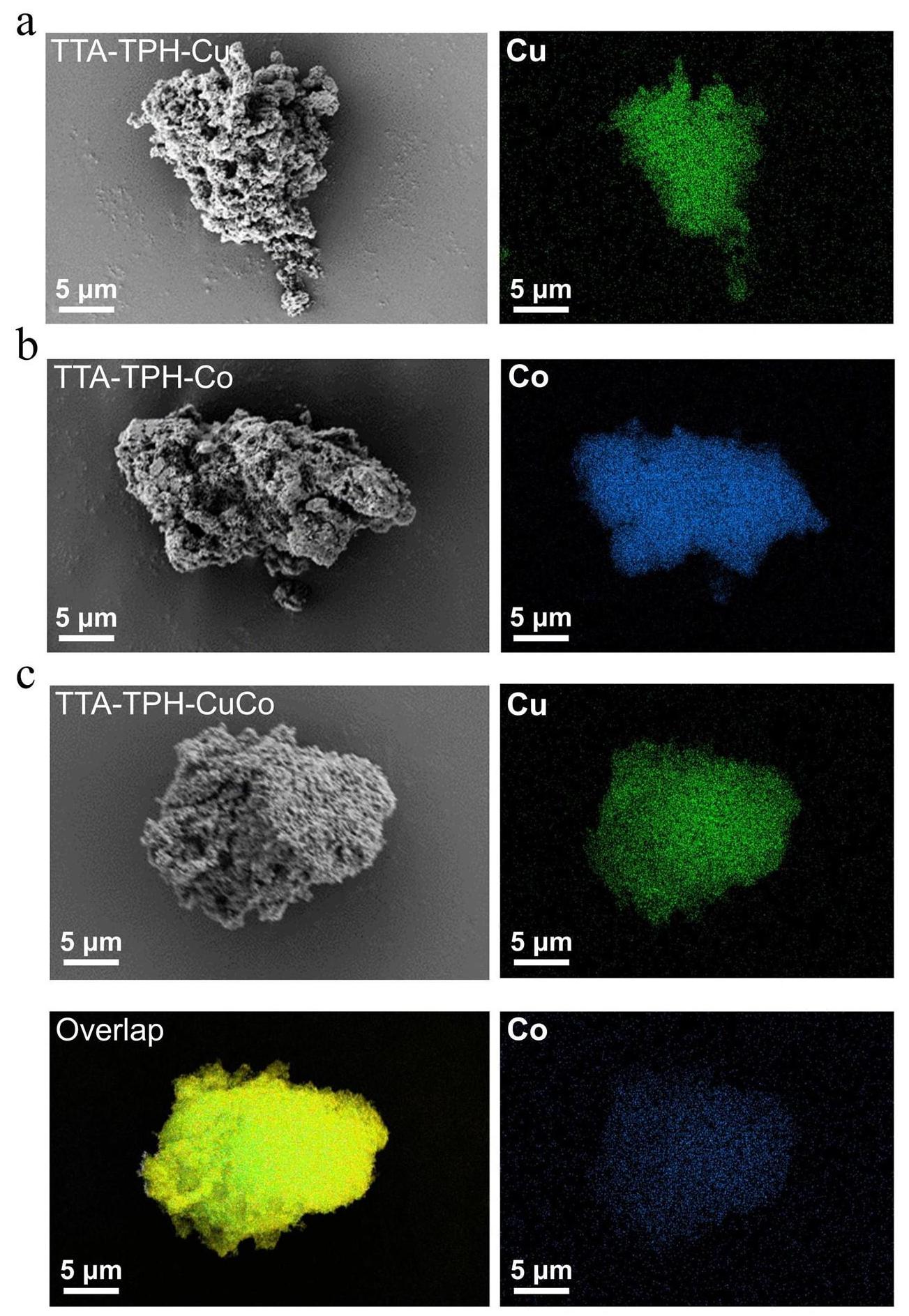


Figure S4. SEM images and the elemental mapping images of (a) TTA-TPH-Cu, (b) TTA-TPH-Co and (c) TTA-TPH-CuCo.


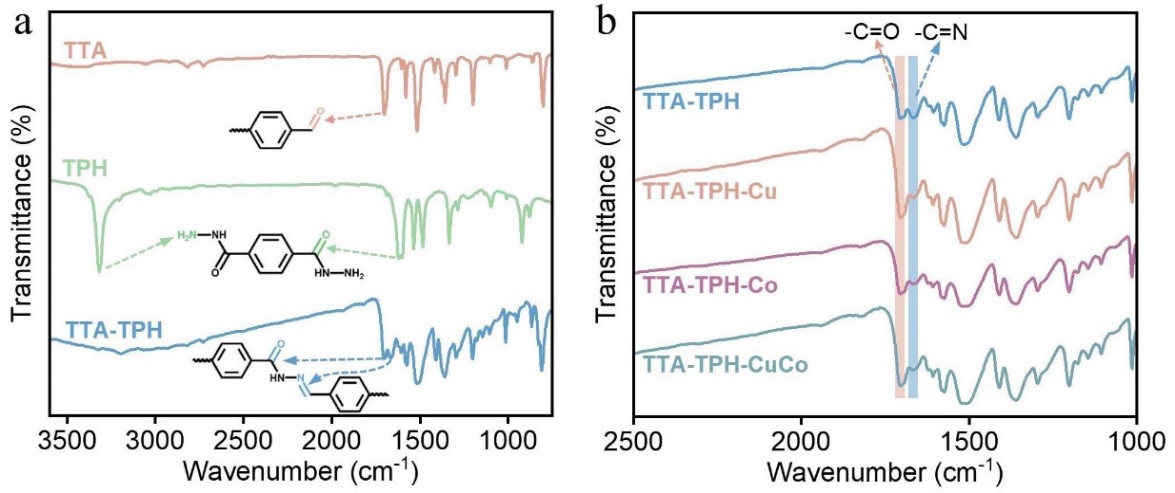


Figure S5. (a) FT-IR spectra of monomers and TTA-TPH. (b) FT-IR spectra of TTA-TPH-Cu, TTA-TPH-Co and TTA-TPH-CuCo.


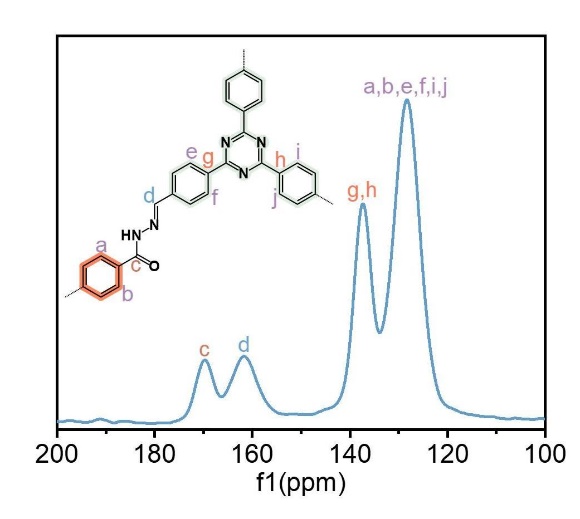


Figure S6. ^13^C solid-state NMR spectrum of TTA-TPH.


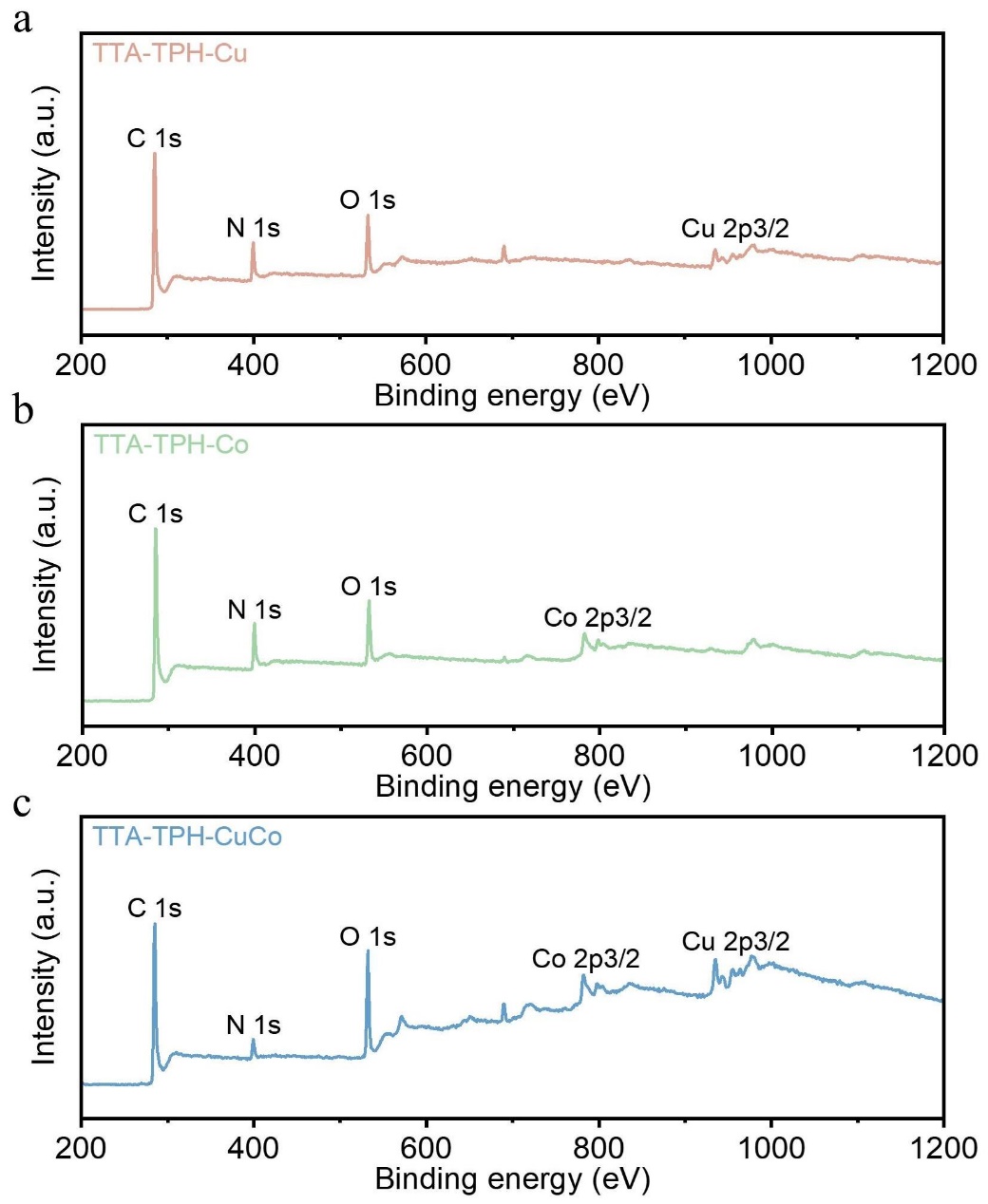


Figure S7. The survey XPS spectra of (a) TTA-TPH-Cu, (b) TTA-TPH-Co and (c)

TTA-TPH-CuCo.


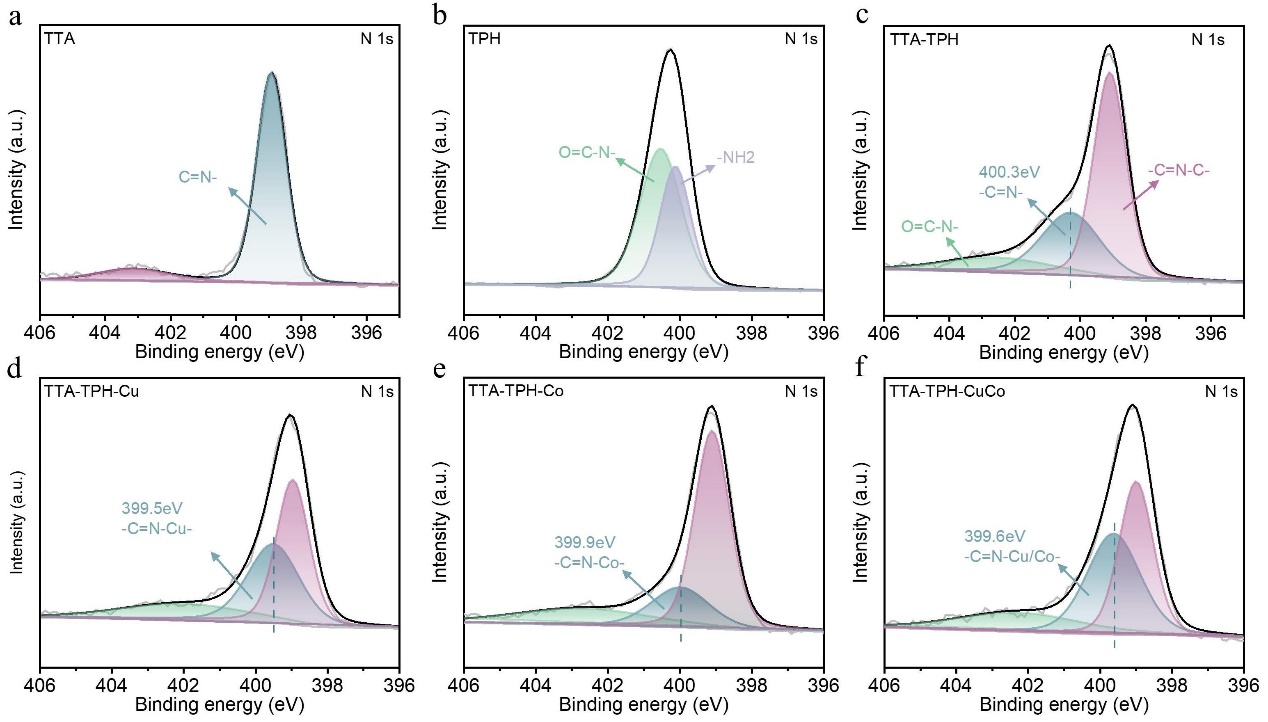


Figure S8. XPS spectra of the N 1s spectra for (a) TTA, (b) TPH, (c)TTA-TPH, (d) TTA-TPH-Cu, (e) TTA-TPH-Co and (f) TTA-TPH-CuCo.


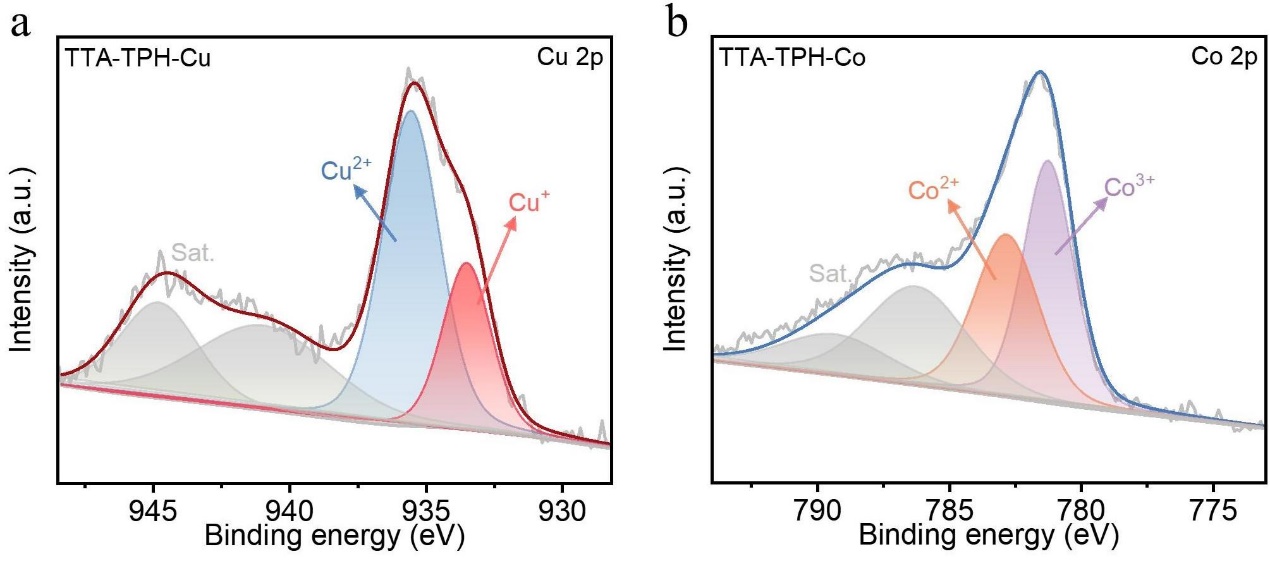


Figure S9. XPS spectra of (a) Cu 2p for TTA-TPH-Cu and (b) Co 2p for TTA-TPH-Co.


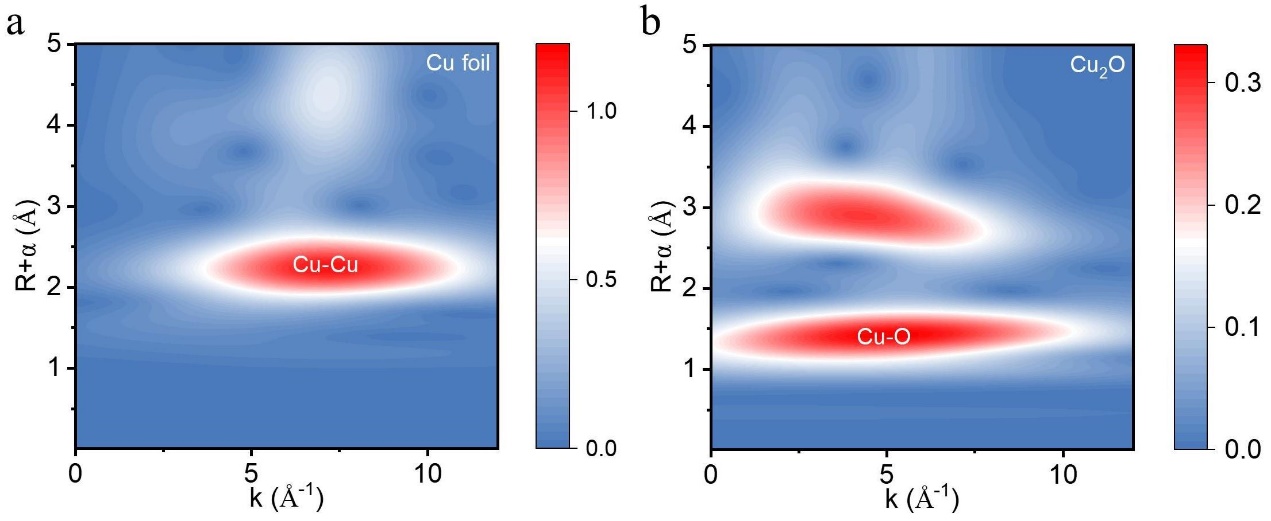


Figure S10. WT-EXAFS plots of Cu for Cu foil and Cu_2_O.


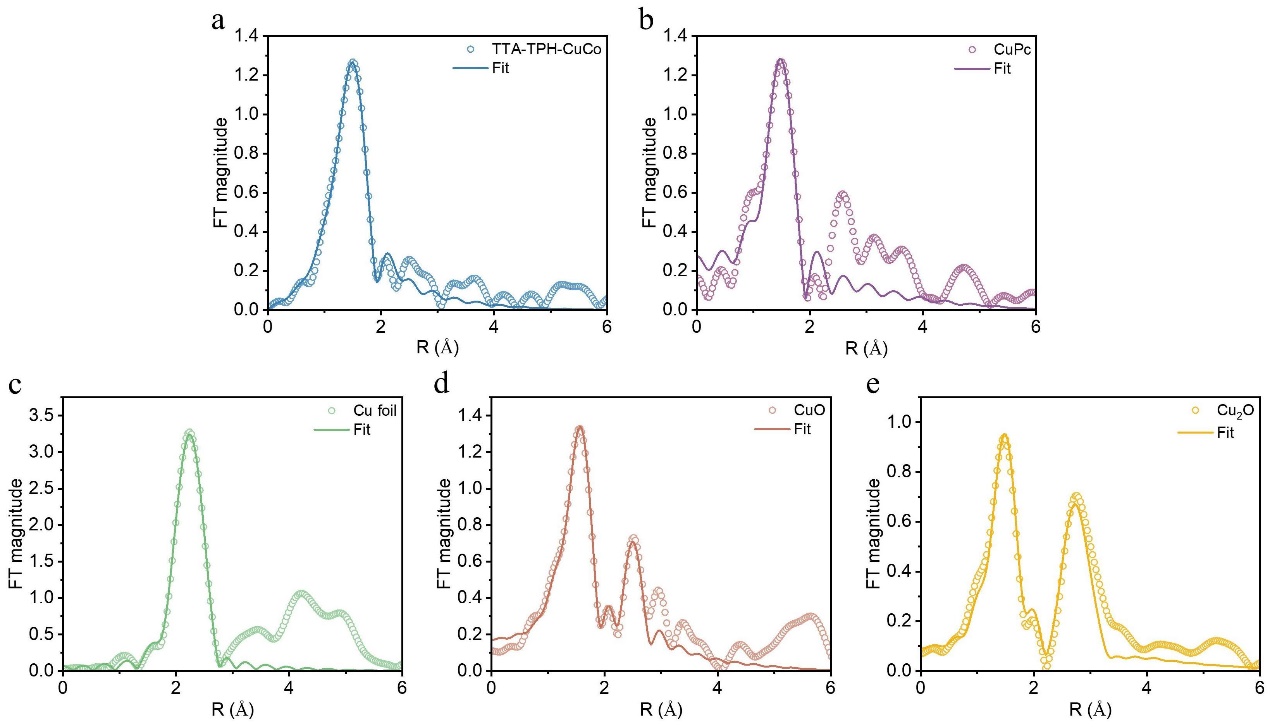
Figure S11. The EXAFS R-space fitting curves of Cu for (a) TTA-TPH-CuCo, (b) CuPc, (c) Cu foil, (d) CuO and (e) Cu_2_O.


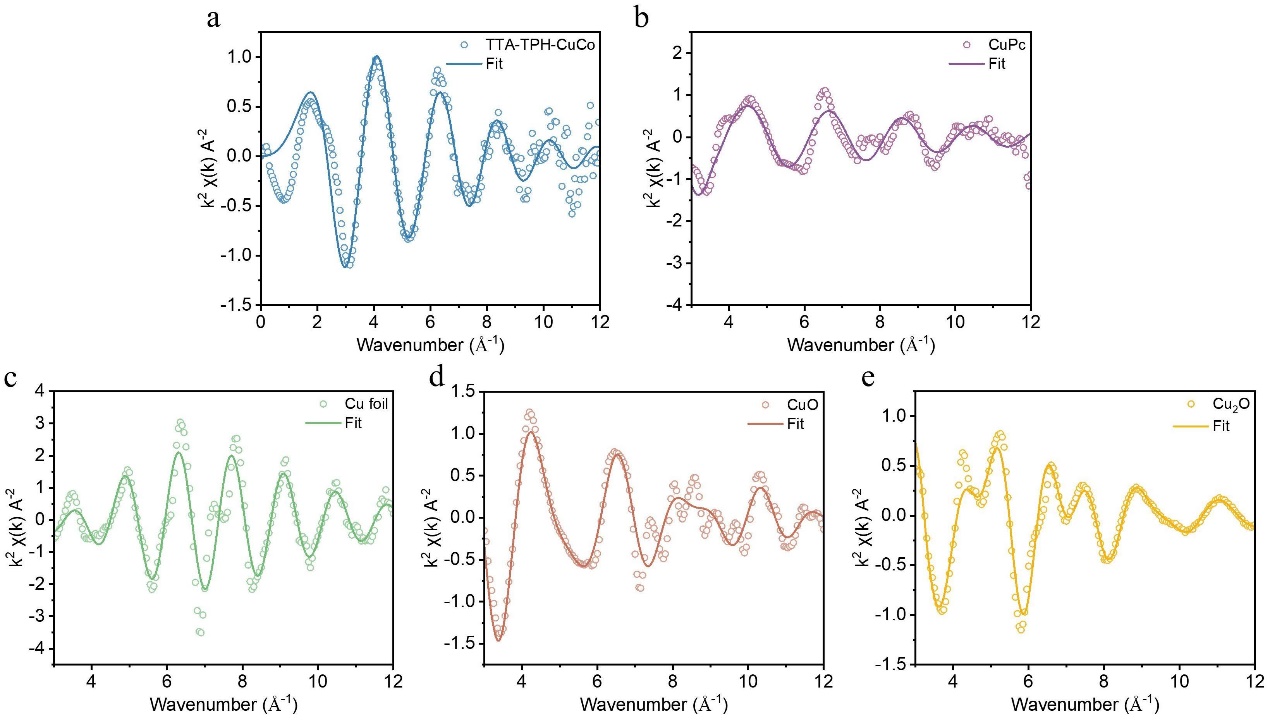


Figure S12. The EXAFS k-space fitting curves of Cu for (a) TTA-TPH-CuCo, (b) CuPc, (c) Cu foil, (d) CuO and (e) Cu_2_O.


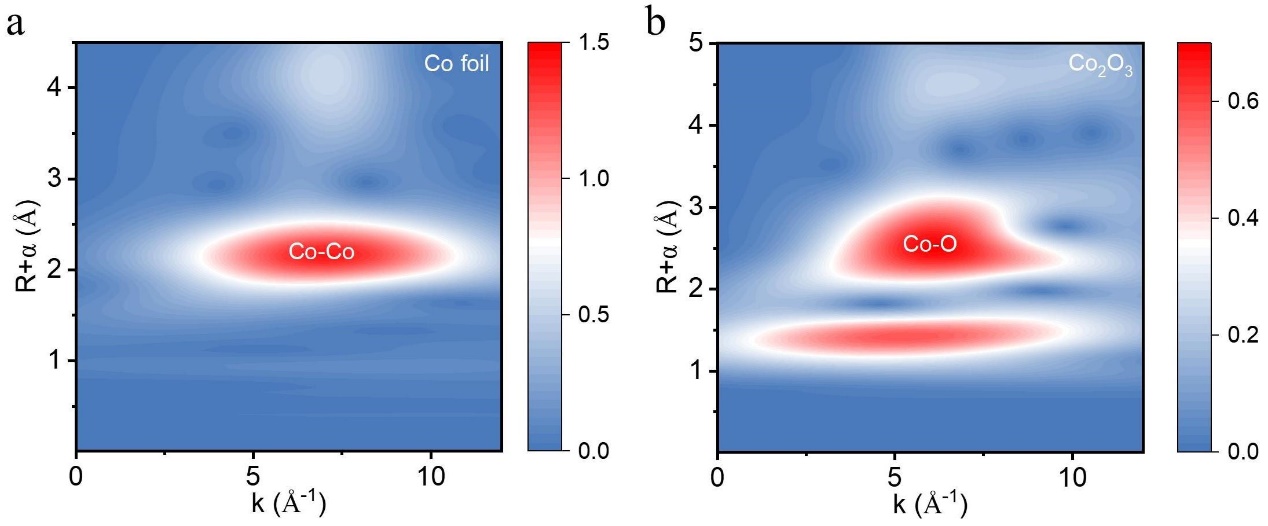
Figure S13. WT-EXAFS plots of Co for Co foil and Co_2_O_3_.


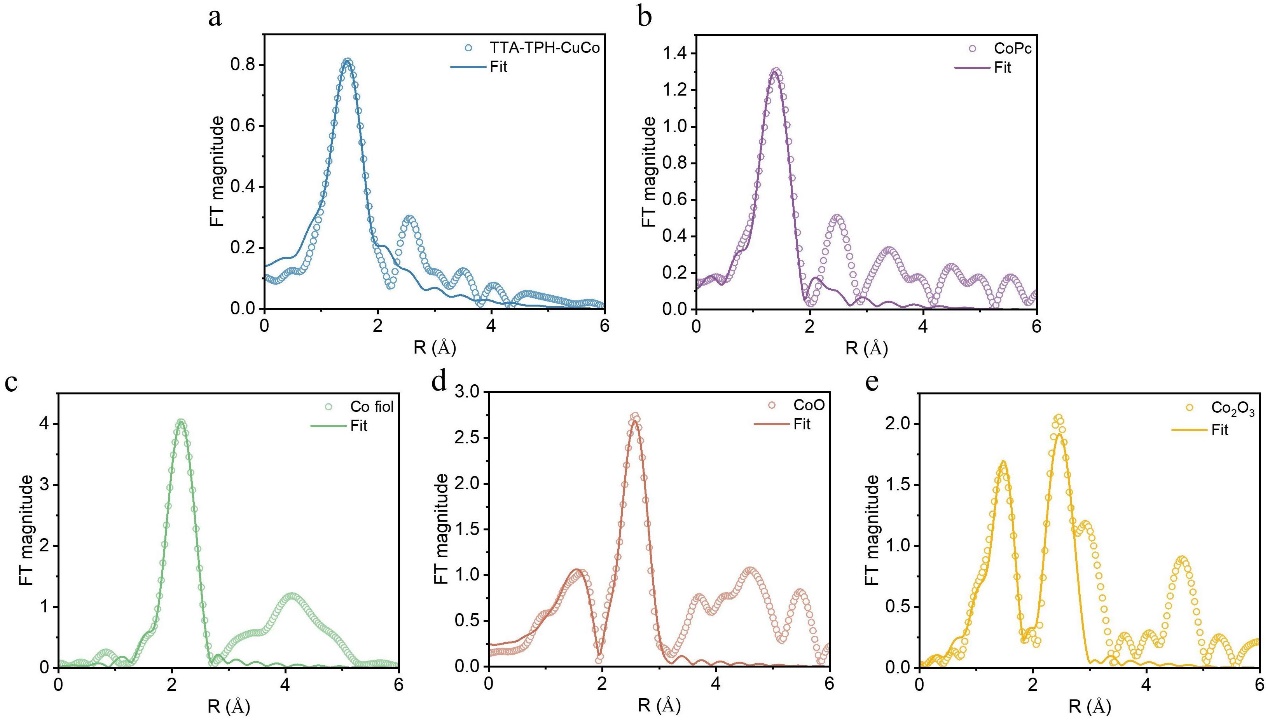
Figure S14. The EXAFS R-space fitting curves of Co for (a) TTA-TPH-CuCo, (b) CoPc, (c) Co foil, (d) CoO and (e) Co_2_O_3_.


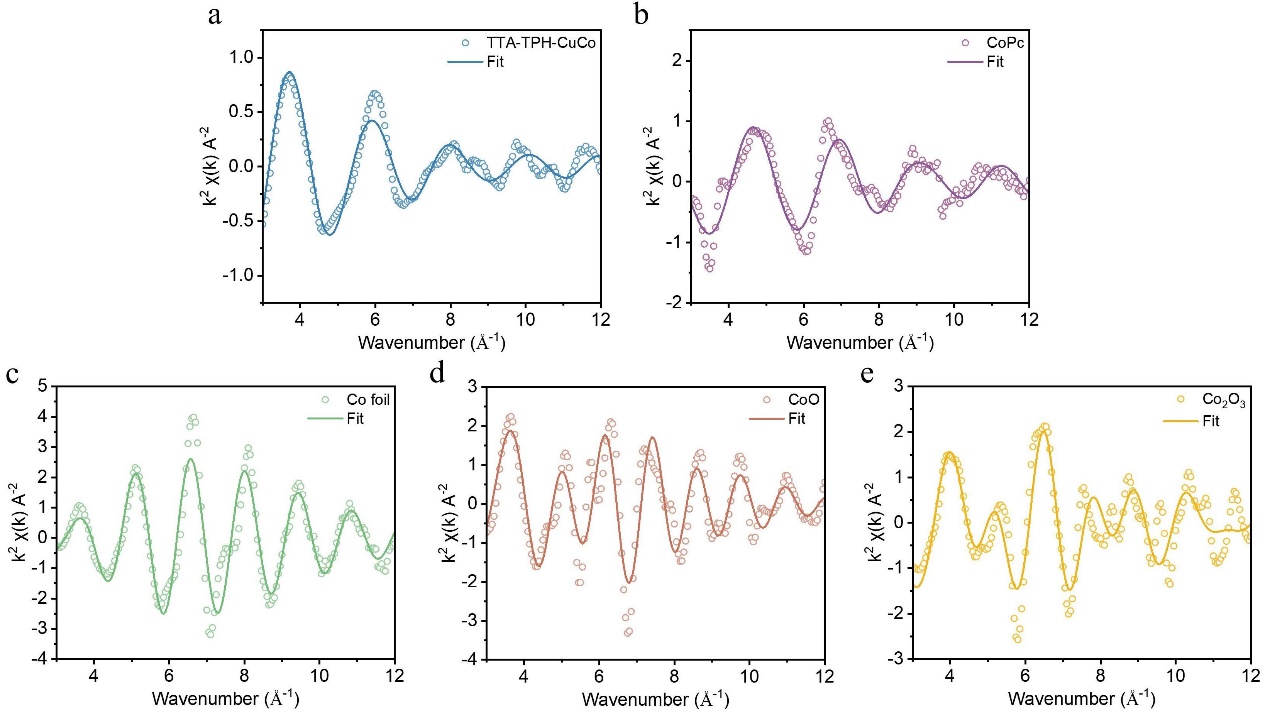
Figure S15. The EXAFS k-space fitting curves of Co for (a) TTA-TPH-CuCo, (b) CoPc, (c) Co foil, (d) CoO and (e) Co_2_O_3_.


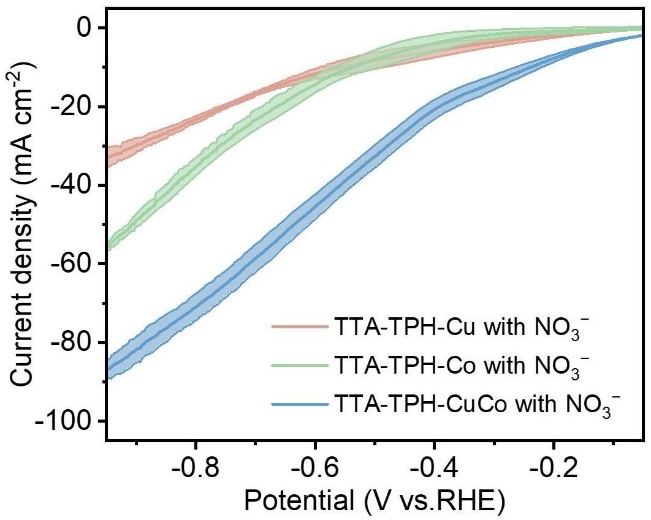


Figure S16. LSV curves in 0.5 M K_2_SO_4_ with 0.1 M NO_3_^−^ (the shaded part represents the errors of three measurements).


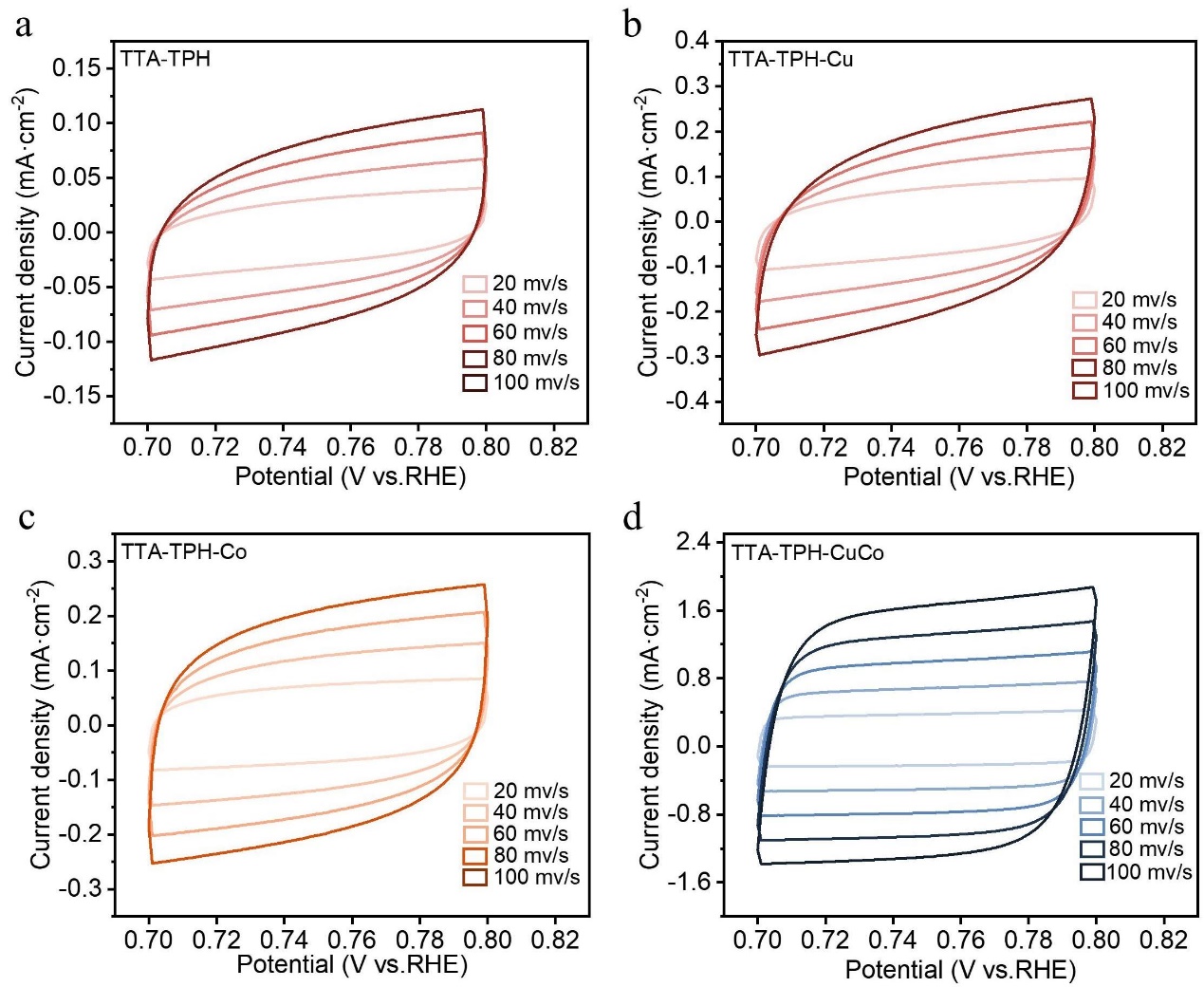
Figure S17. CV curves of (a) TTA-TPH, (b) TTA-TPH-Cu, (c) TTA-TPH-Co, and (d) TTA-TPH-CuCo at varying scan rates within the non-Faradaic region.


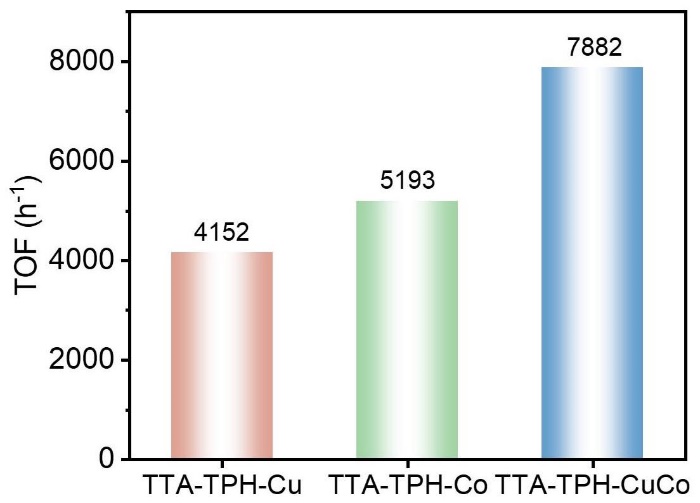


Figure S18. The turnover frequency of TTA-TPH-CuCo, TTA-TPH-Cu and TTA-TPH-Co.


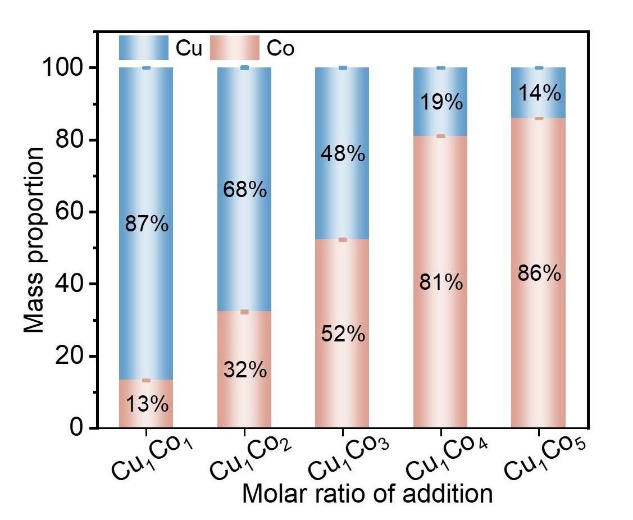


Figure S19. The actual mass proportions of CuCo under different addition ratios.


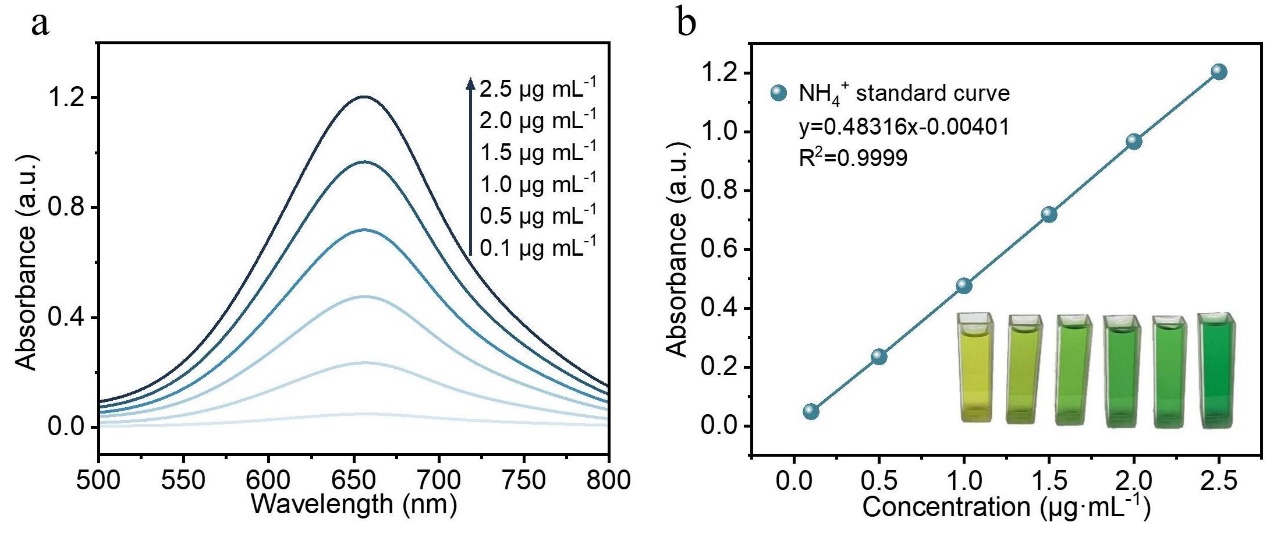


Figure S20. Determination of NH_3_. (a) UV-Vis absorption spectra and (b) the standard curve of NH_4_^+^ for different concentrations.


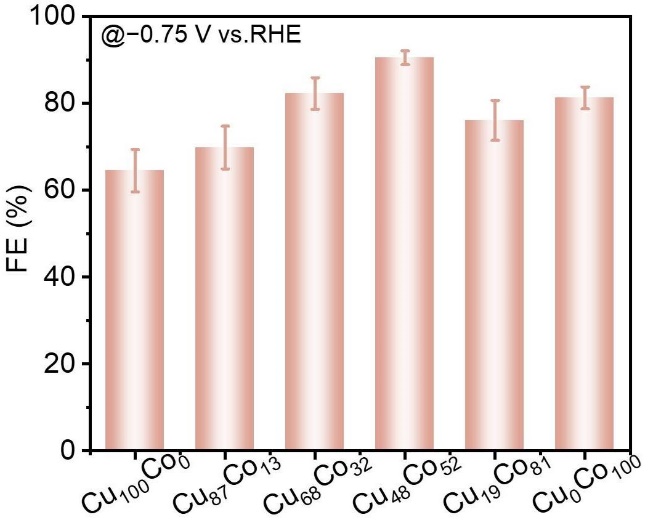


Figure S21. NH_3_-FE at different CuCo proportions.


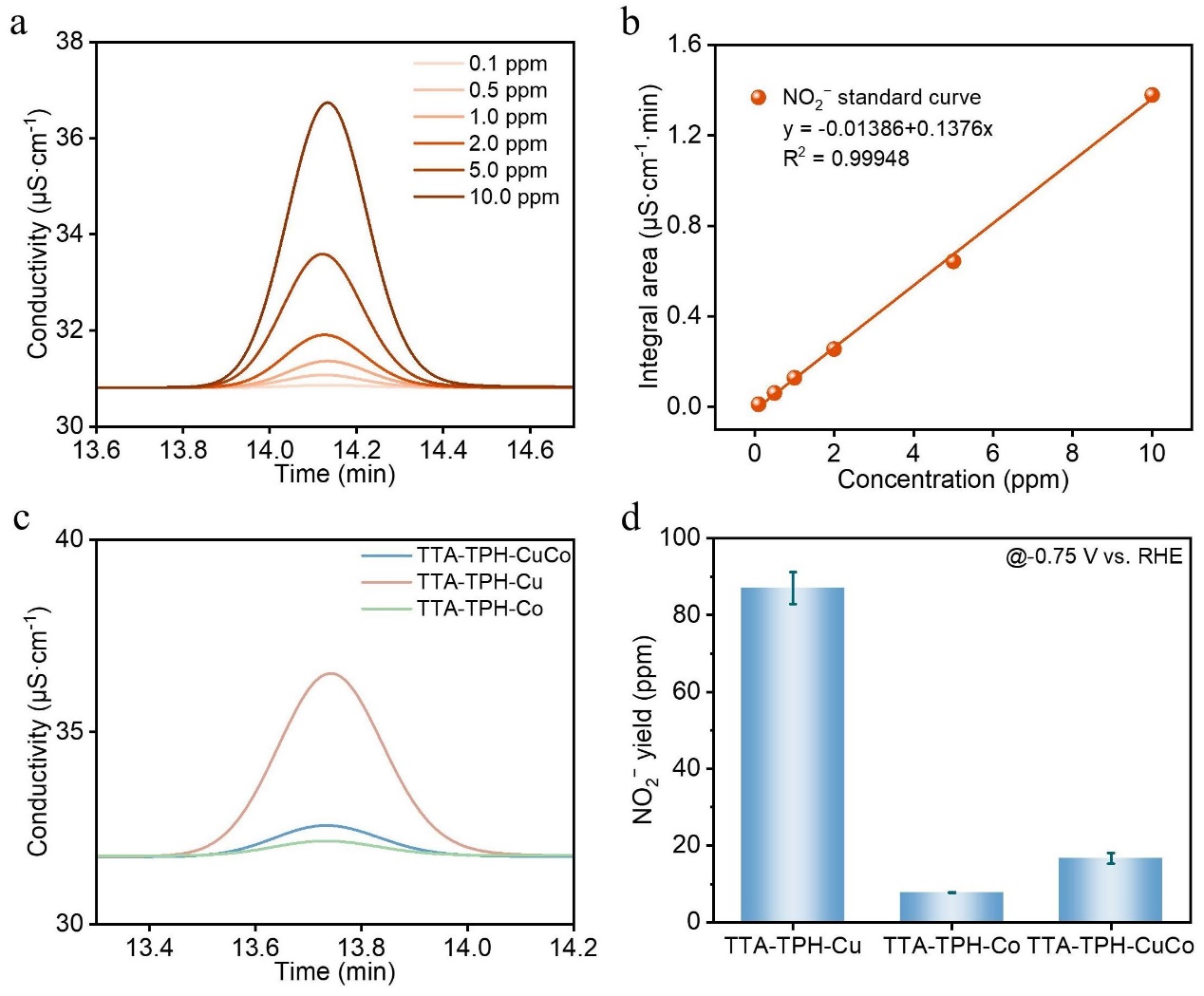


Figure S22. Determination of nitrite (NO_2_^−^). (a) The conductivity-time spectrograms of anion chromatography and (b) the standard curve of NO_2_^−^ for different concentrations. (c) The conductivity-time spectrograms and (d) NO_2_^−^ yields of TTA-TPH-Cu, TTA-TPH-Co and TTA-TPH-CuCo.


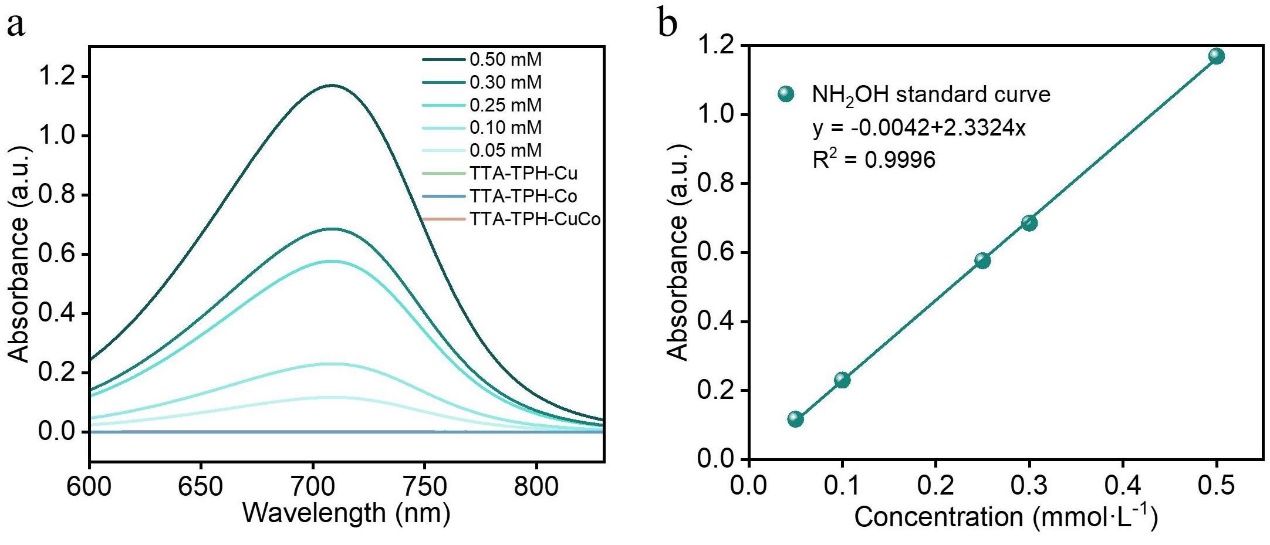
Figure S23. Determination of hydroxylamine (NH_2_OH). (a) The UV-Vis absorption spectra and (b) the standard curve of NH_2_OH for different concentrations.


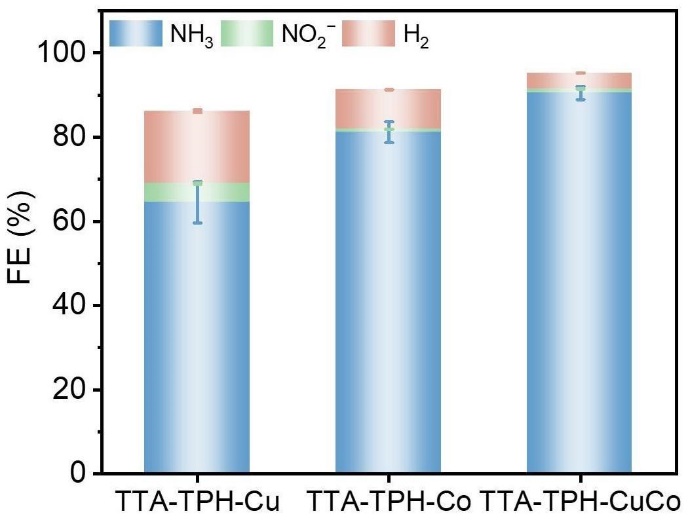


Figure S24. FE of NH_3_, NO_2_^−^ and H_2_ over TTA-TPH-CuCo, TTA-TPH-Cu and TTA-TPH-Co at −0.75 V vs. RHE.


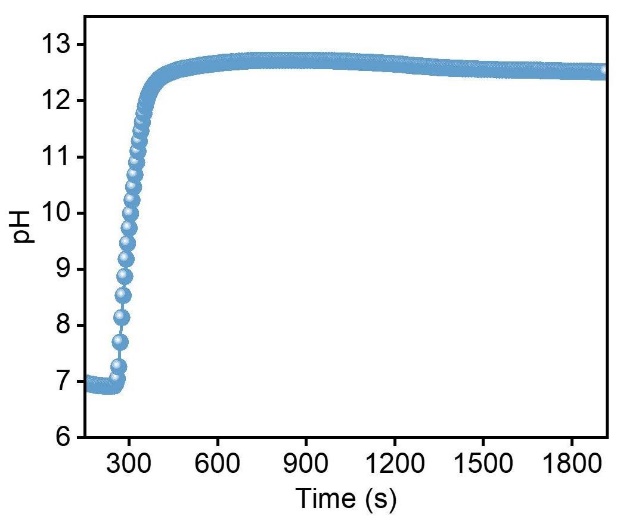


Figure S25. The pH curve of TTA-TPH-CuCo during the 30 minutes electrochemical test in a solution containing 0.1 M NO_3_^−^ and 0.5 M K_2_SO_4_ at −0.75 V vs. RHE.


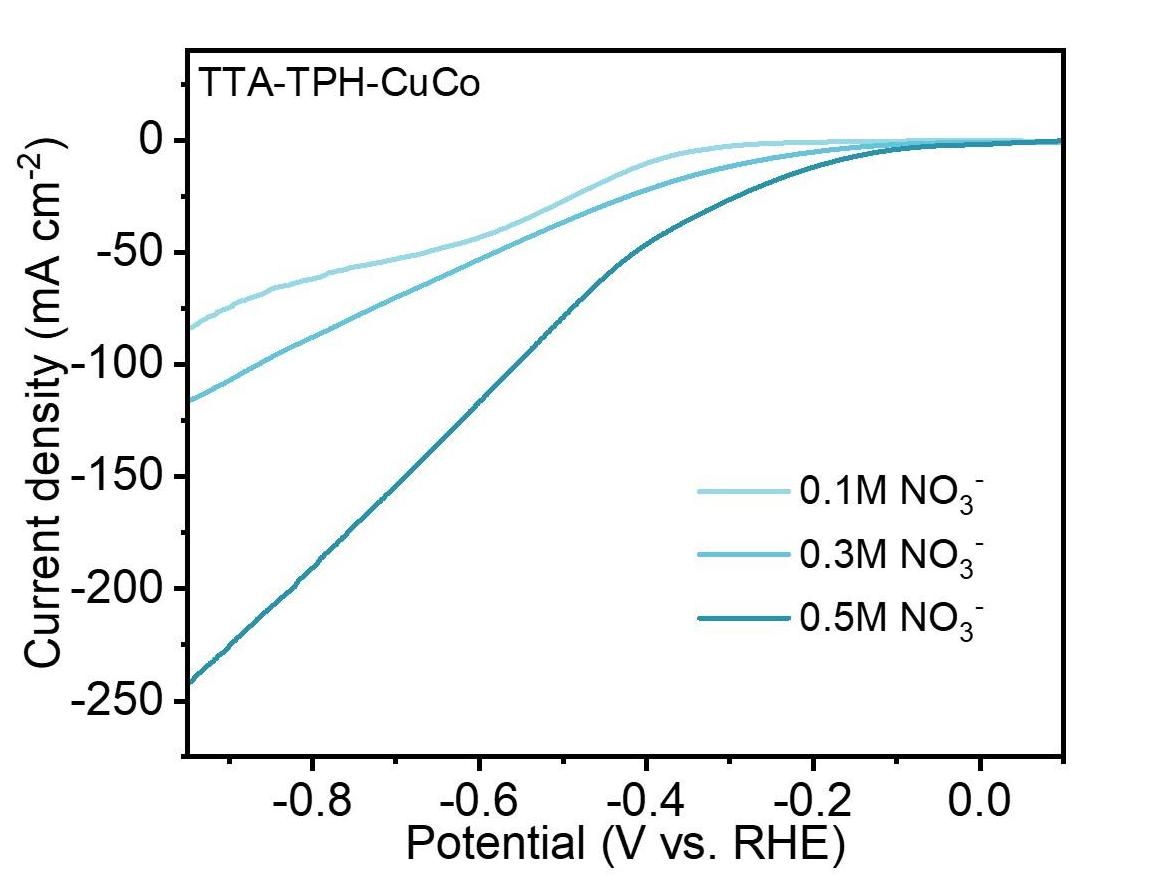


Figure S26. The LSV curves of TTA-TPH-CuCo with different NO_3_^−^ concentrations.


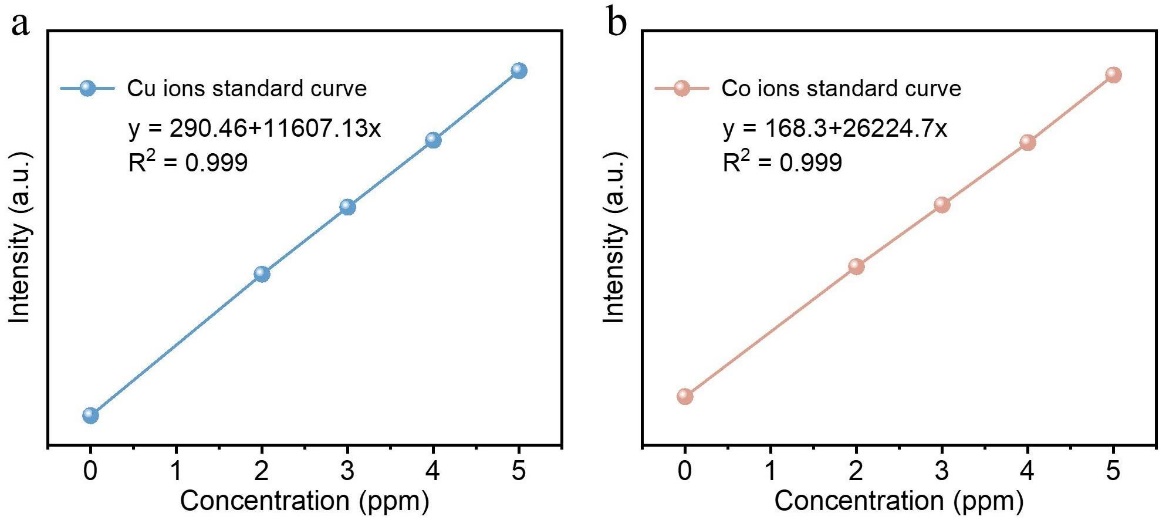


Figure S27. The standard ICP curves of (a) Cu ions and (b) Co ions at different concentrations.


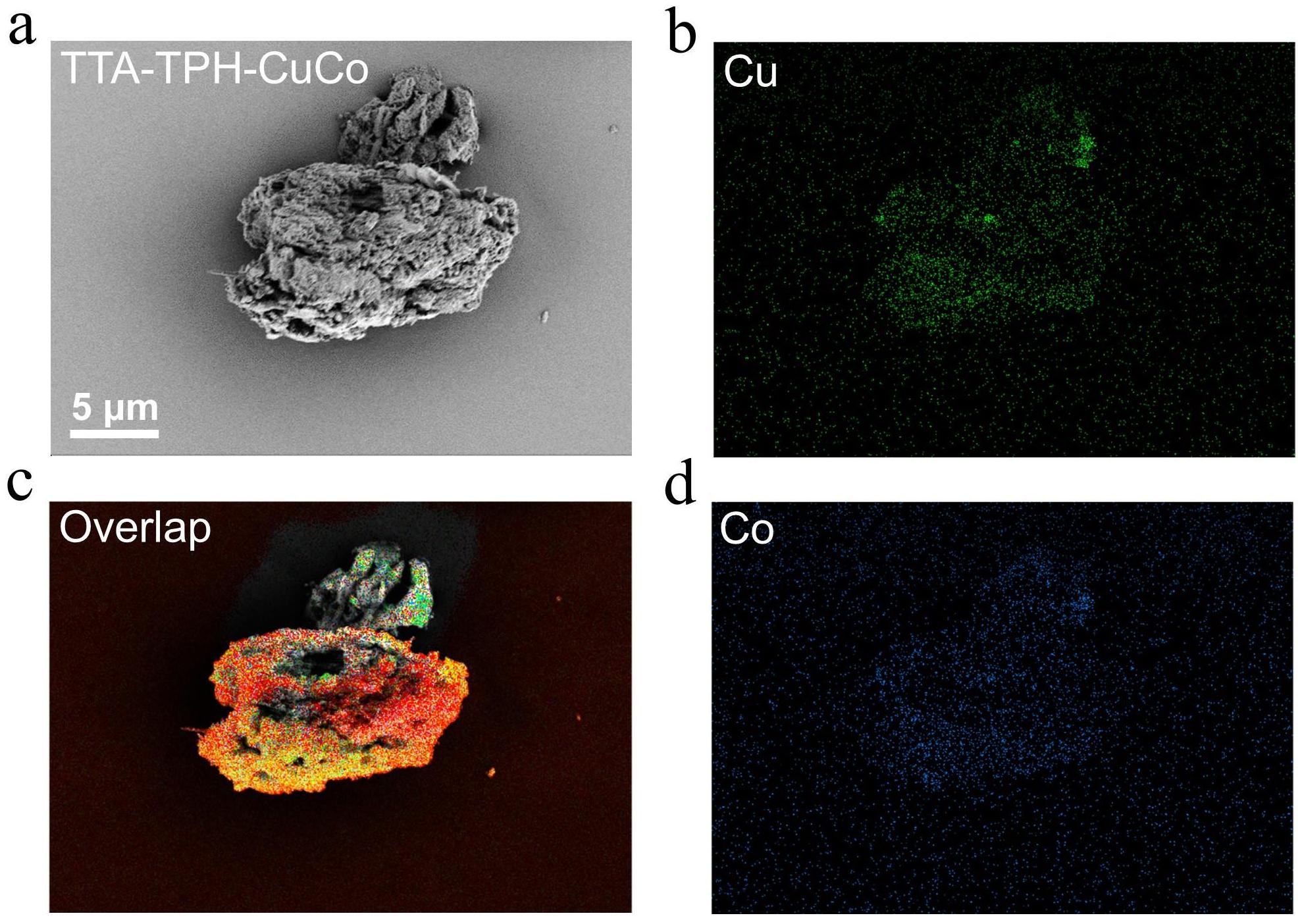


Figure S28. SEM image and the elemental mapping images of TTA-TPH-CuCo after the NO_3_RR process.


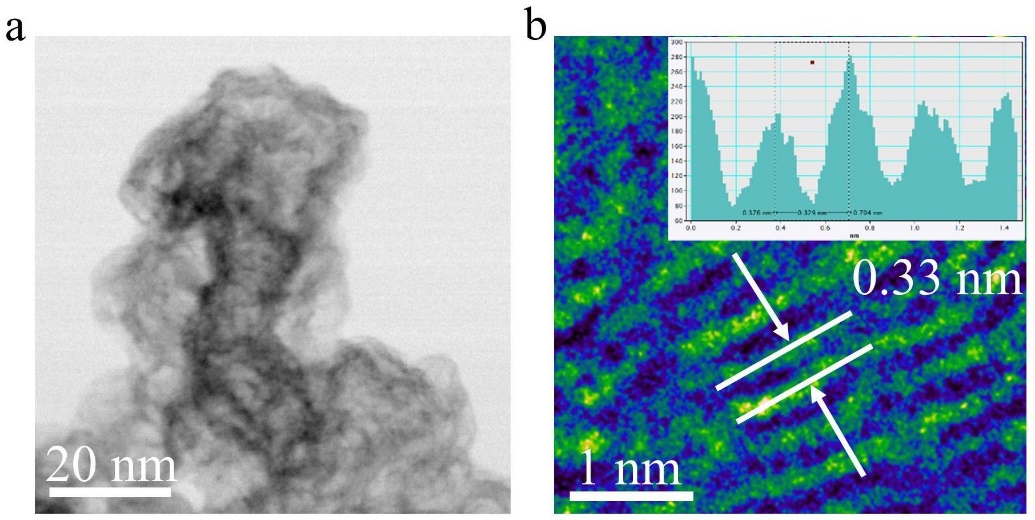


Figure S29. (a) TEM and (b) HR-TEM images of TTA-TPH-CuCo after electrochemical test, the inset shows the distance of (001) lattice plane.


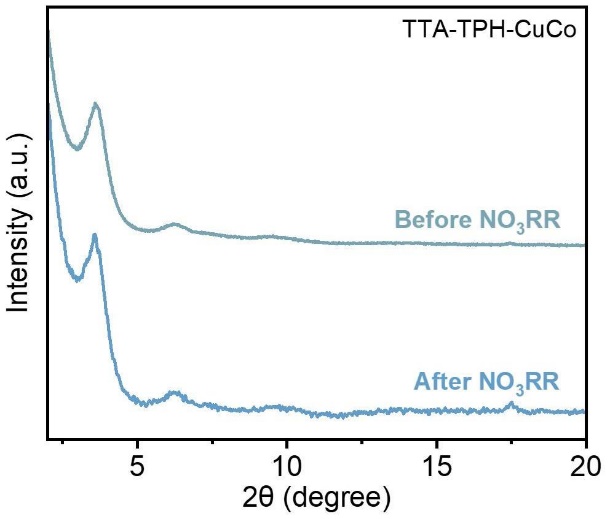


Figure S30. PXRD pattern of TTA-TPH-CuCo before and after NO_3_RR process.


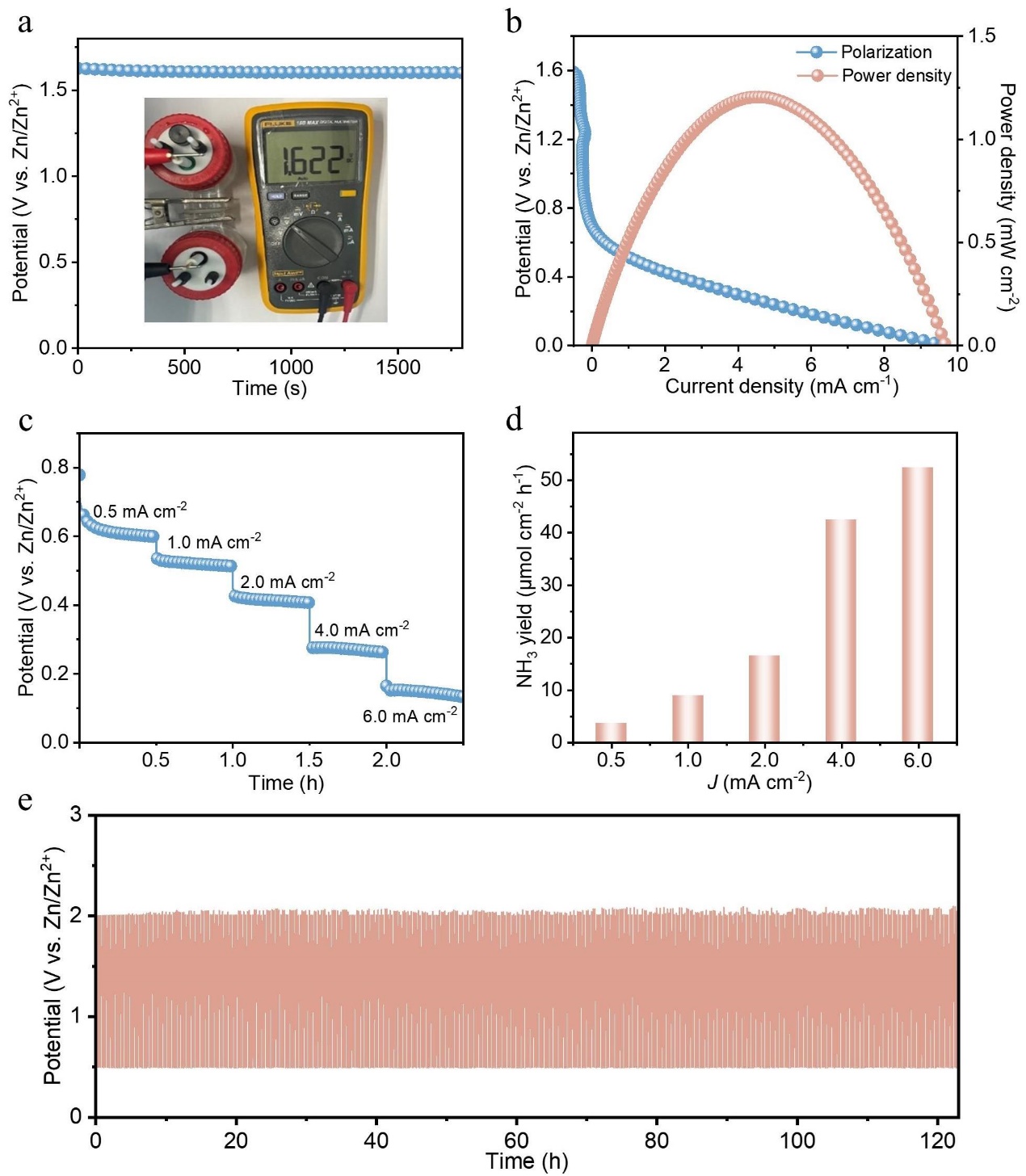


Figure S31. Zn-NO_3_^−^ battery. (a) Open circuit voltage of TTA-TPH-CuCo based Zn-NO_3_^−^ battery, (b) Polarization curve and power density of assembled Zn-NO_3_^−^ battery, (c) Discharge curves under different current densities. (d) The corresponding NH_3_ yield under different current densities.


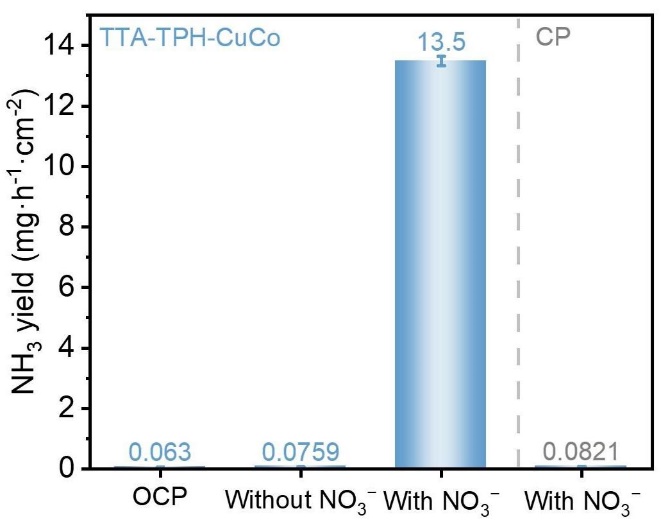


Figure S32. NO_3_RR performance over TTA-TPH-CuCo and carbon paper (CP) under different conditions (OCP represents open circuit potential).


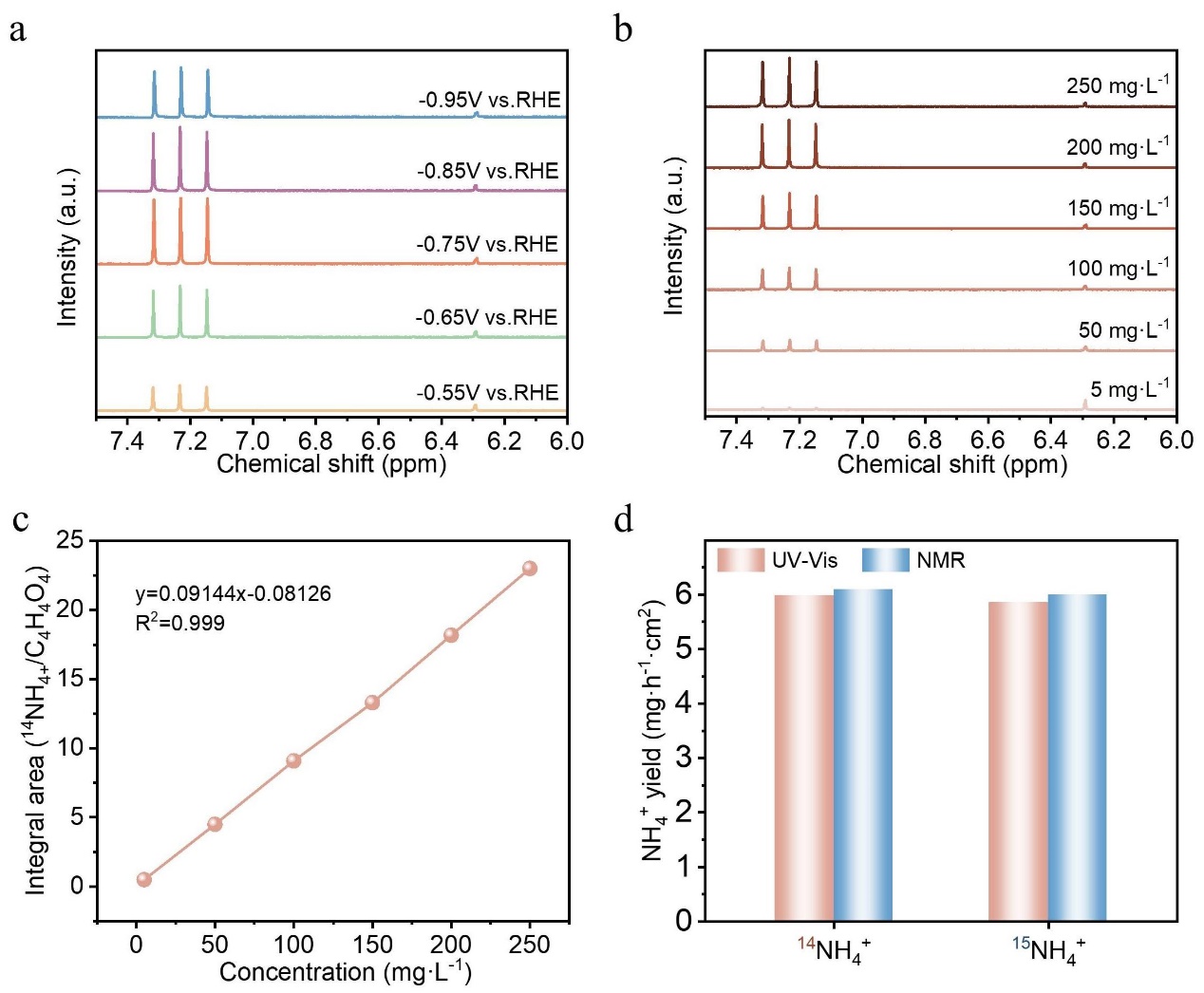


Figure S33. (a) The ^1^H NMR signals of the collected electrolytes tested at different potentials. (b) The ^1^H NMR spectra and (c) calibration curves of standard NH_4_^+^ solutions. (d) The comparison of NH_3_ yield and FE using UV-Vis and NMR methods.


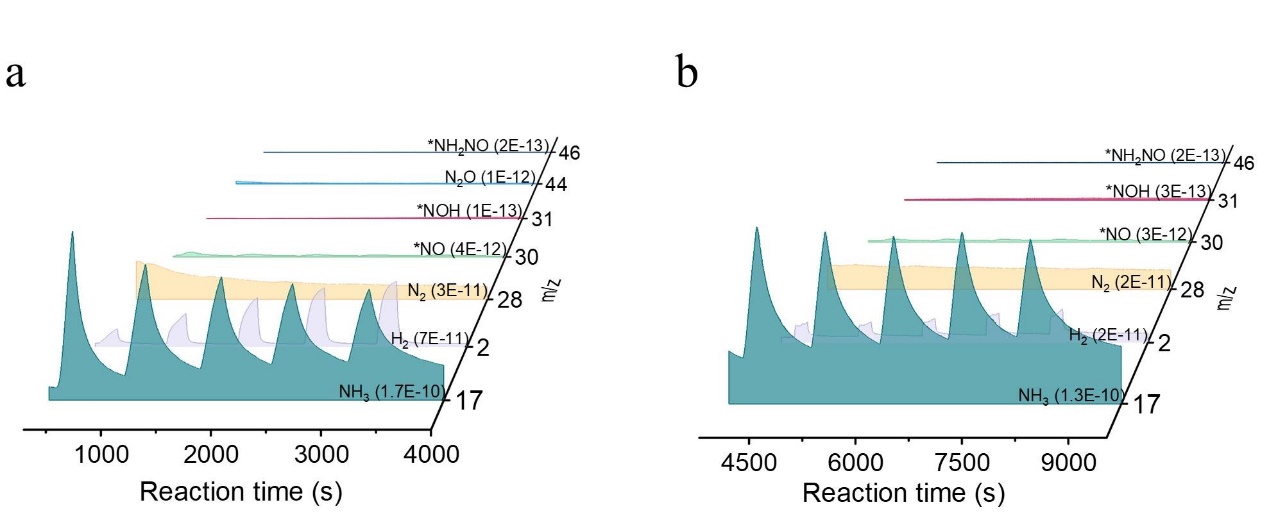


Figure S34. *In situ* DEMS patterns of (a) TTA-TPH-Cu and (b) TTA-TPH-Co.

**
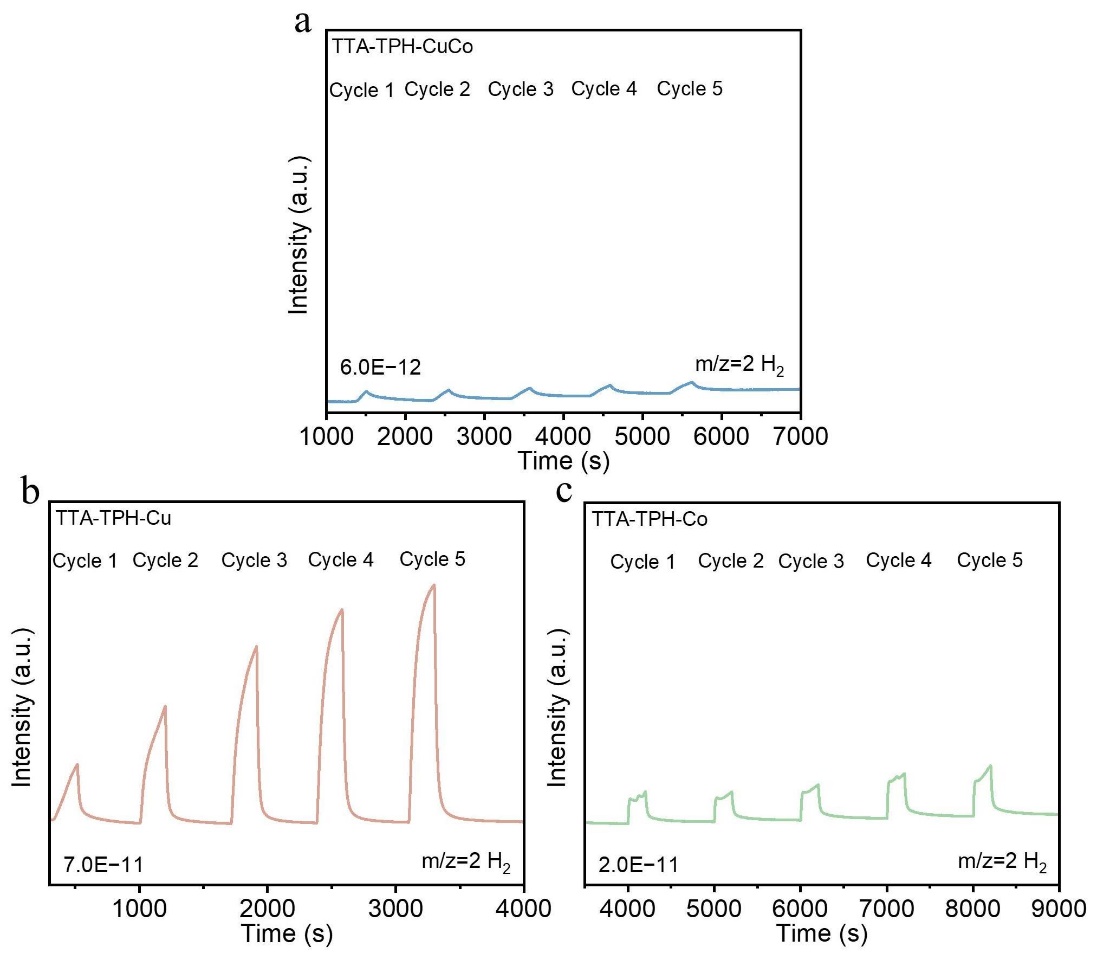
**

Figure S35. *In situ* DEMS of H_2_ signal for (a) TTA-TPH-CuCo, (a) TTA-TPH-Cu and (c) TTA-TPH-Co.


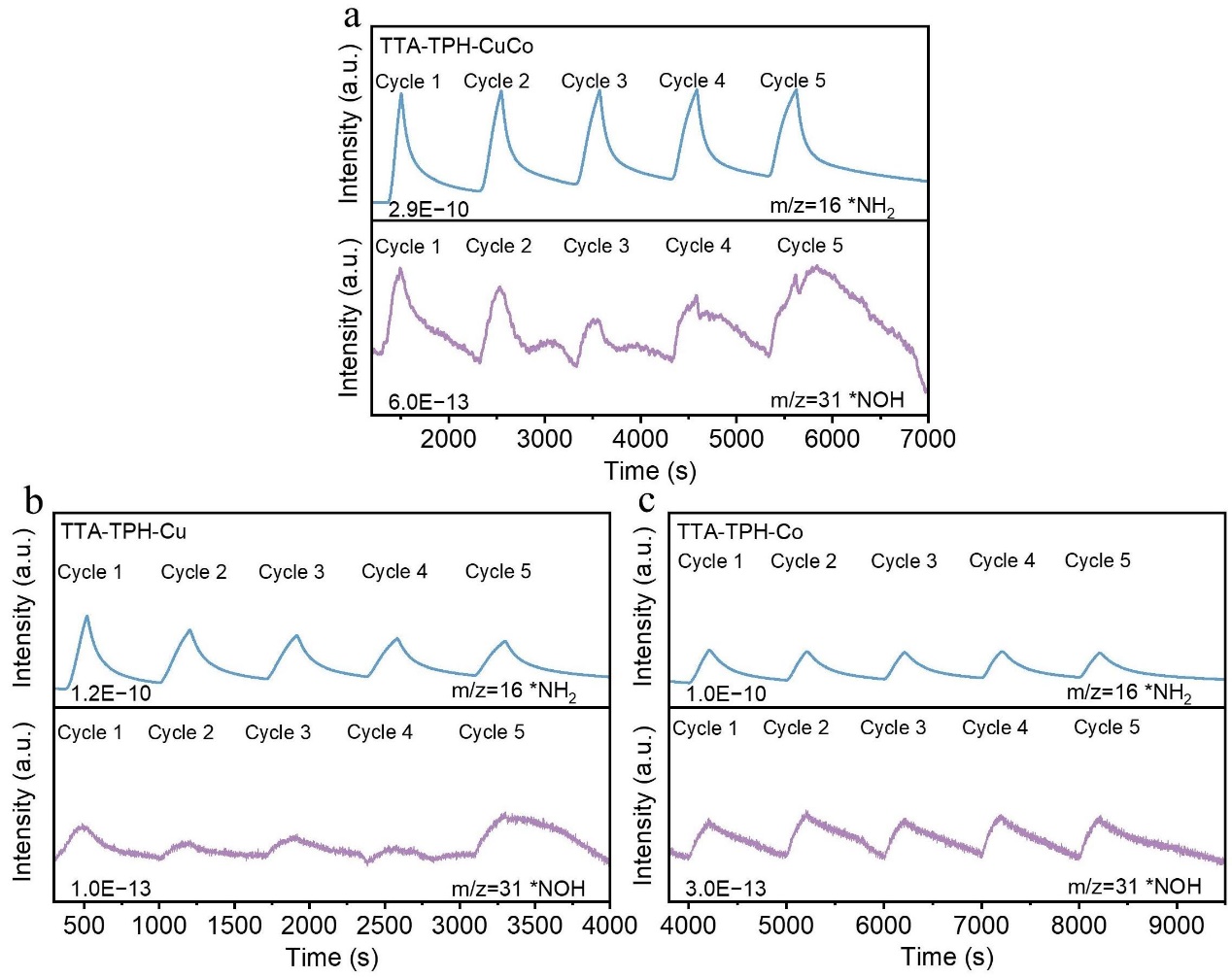


Figure S36. *In situ* DEMS of *NOH and *NH_2_ signal for (a) TTA-TPH-CuCo, (b) TTA-TPH-Cu and (c) TTA-TPH-Co.


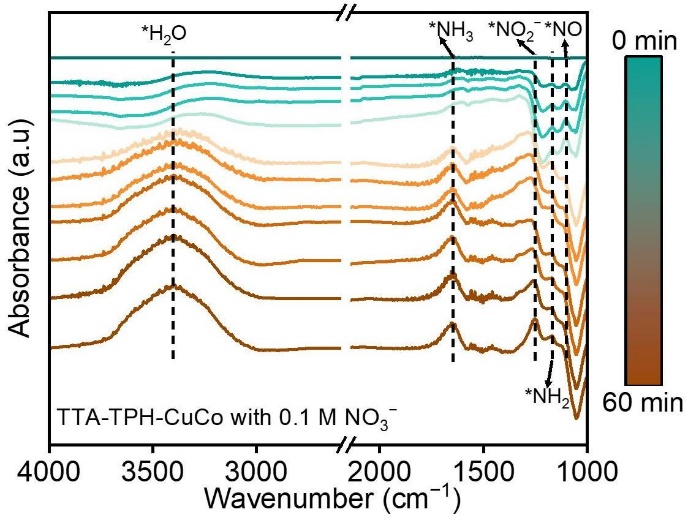


Figure S37. *In situ* ATR-IRAS measurements during 60 minutes test (at −0.75 V vs. RHE).


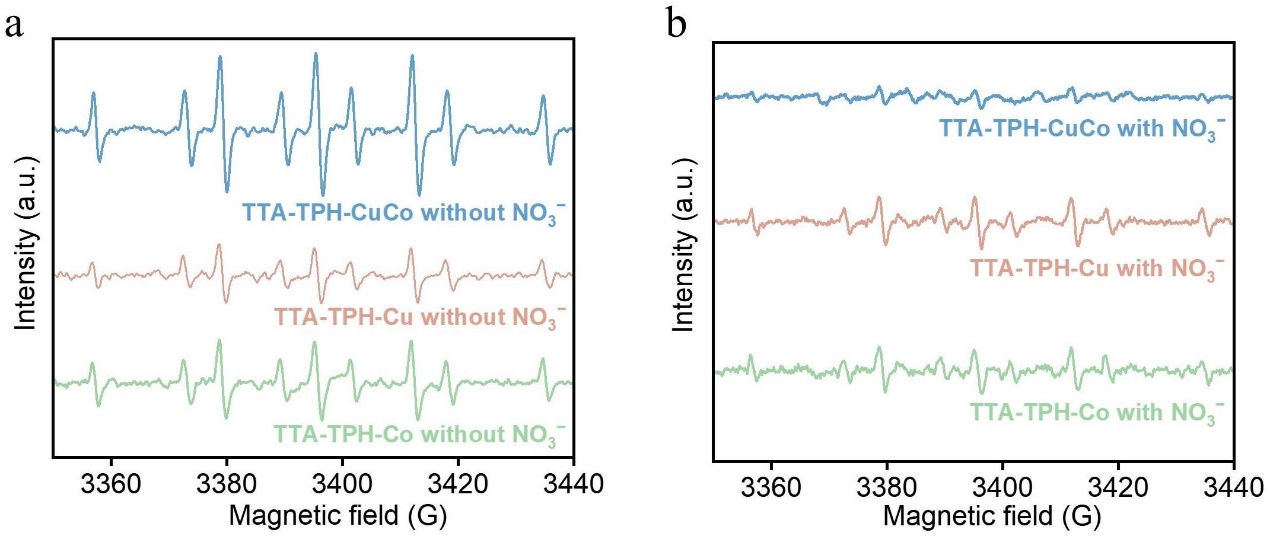
Figure S38. EPR spectra (a) with and (b) without 0.1 M NO_3_^−^.


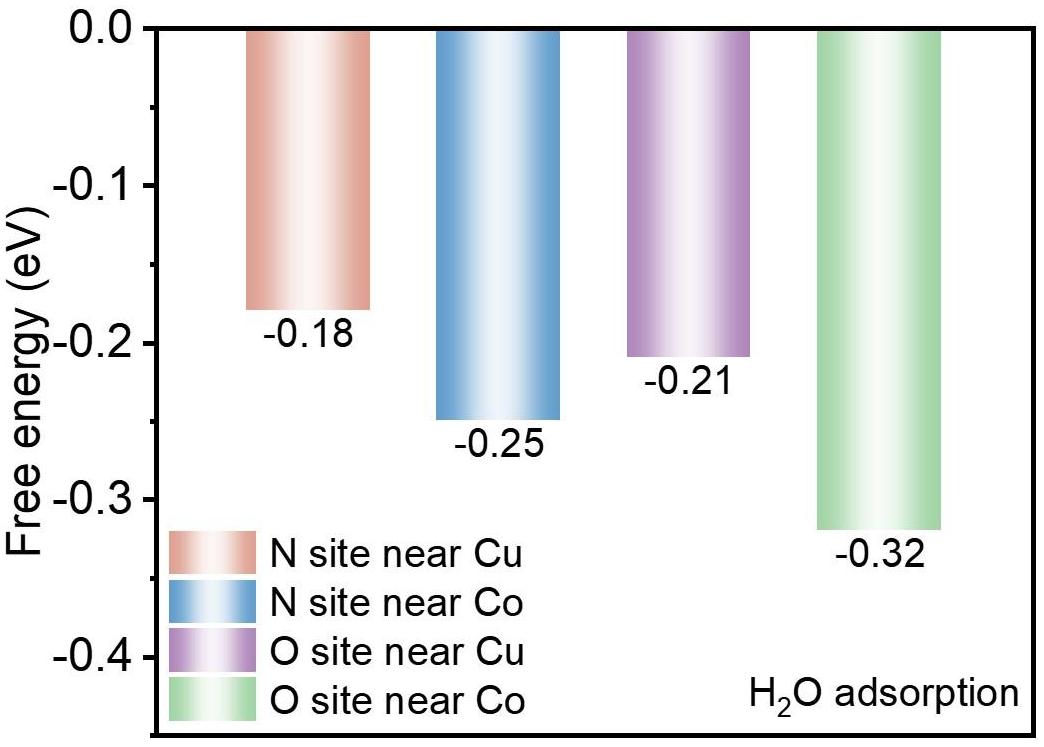


Figure S39. Energy barriers during the processes of H_2_O adsorption.


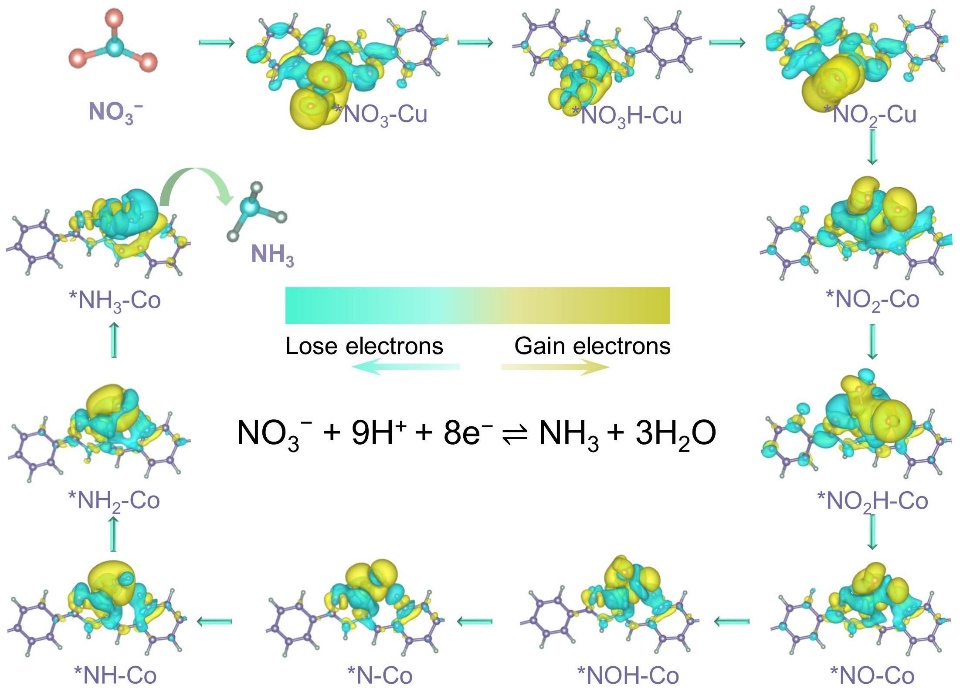


Figure S40. The electrons transfer process during the NO_3_RR process (cyan represents loss of electrons while yellow represents gain of electrons).

**Table S1**. Atomic coordinates of the AA-stacking mode of TTA-TPH-COF.

| Space group: *P*6  *a* = *b* = 28.98 and *c* = 3.37 Å  *α* =*β* = 90° and *γ* = 120° | | | |
| --- | --- | --- | --- |
|  | X | Y | Z |
| C1 | 0.31093 | 0.67783 | -0.5 |
| C2 | 0.2991 | 0.64381 | -0.5 |
| C3 | 0.24844 | 0.5831 | -0.5 |
| C4 | 0.26122 | 0.61853 | -0.5 |
| C5 | 0.23686 | 0.62917 | -0.5 |
| C6 | 0.20222 | 0.60596 | -0.5 |
| C7 | 0.19037 | 0.5712 | -0.5 |
| C8 | 0.21376 | 0.56005 | -0.5 |
| C9 | 0.15389 | 0.54625 | -0.5 |
| C10 | 0.97903 | 0.5141 | -0.5 |
| C11 | 0.01412 | 0.53497 | -0.5 |
| C12 | 0.0731 | 0.54456 | -0.5 |
| O13 | 0.08413 | 0.57502 | -0.5 |
| C14 | 0.96427 | 0.47869 | 0.5 |
| N15 | 0.09672 | 0.53252 | 0.5 |
| N16 | 0.86846 | 0.44417 | 0.5 |
| H17 | 0.60764 | 0.29393 | 0.5 |
| H18 | 0.73576 | 0.42812 | 0.5 |
| H19 | 0.75671 | 0.34477 | 0.5 |
| H20 | 0.81532 | 0.38462 | 0.5 |
| H21 | 0.79483 | 0.46676 | 0.5 |
| H22 | 0.8542 | 0.48047 | 0.5 |
| H23 | 0.03592 | 0.47367 | 0.5 |
| H24 | 0.97565 | 0.43794 | 0.5 |
| H25 | 0.08864 | 0.50679 | 0.5 |
|  |  |  |  |

**Table S2**. EXAFS fitting parameters at the Cu K-edge for Cu foil, CuO, Cu_2_O, CuPc and TTA-TPH-CuCo.

| Sample | Shell | *CN^a^* | *R*(Å)*^b^* | σ2 (Å^2^) ^c^ | Δ*E*_0_(eV)*^d^* | R factor |
| --- | --- | --- | --- | --- | --- | --- |
| Cu foil | Cu-Cu | 11.60±0.72 | 2.54±0.01 | 0.008±0.001 | 4.10±0.63 | 0.004 |
| CuO | Cu-O | 3.81±0.50 | 1.96±0.01 | 0.003±0.001 | 10.09±1.13 | 0.020 |
|  | Cu-O | 2.22±0.73 | 2.87±0.01 | 0.001±0.001 |  |  |
| Cu_2_O | Cu-O | 4.24±0.47 | 1.85±0.01 | 0.002±0.001 | 11.52±0.86 | 0.010 |
|  | Cu-Cu | 7.46±0.25 | 3.01±0.01 | 0.024±0.003 |  |  |
| CuPc | Cu-N | 3.37±0.89 | 1.96±0.06 | 0.003±0.021 | 13.11±0.39 | 0.035 |
|  | Cu-C | 3.00±0.95 | 2.32±0.12 | 0.038±0.018 |  |  |
| TTA-TPH-CuCo | Cu-N | 1.95±0.39 | 1.99±0.08 | 0.001±0.002 | -2.20±1.59 | 0.015 |
|  | Cu-O | 1.00±0.49 | 1.85±0.05 | 0.002±0.005 |  |  |

*^a^CN*, coordination number; *^b^R*, distance between absorber and backscatter atoms; *^c^σ*^2^, Debye-Waller factor to account for both thermal and structural disorders; *^d^ΔE*_0_, inner potential correction; *R* factor indicates the goodness of the fit.

**Table S3**. EXAFS fitting parameters at the Co K-edge for Co foil, CoO, Co_2_O_3_, CoPc and TTA-TPH-CuCo.

| Sample | Shell | *CN^a^* | *R*(Å)*^b^* | σ^2^ (Å^2^) ^c^ | Δ*E*_0_(eV)*^d^* | R factor |
| --- | --- | --- | --- | --- | --- | --- |
| Co foil | Co-Co | 11.39±0.58 | 2.49±0.01 | 0.006±0.001 | 8.33±0.53 | 0.002 |
| CoO | Co-O | 5.36±0.59 | 2.10±0.01 | 0.003±0.001 | -5.47±0.78 | 0.006 |
|  | Co-Co | 11.40±1.24 | 3.00±0.01 | 0.001±0.001 |  |  |
| Co_2_O_3_ | Co-O | 5.70±0.42 | 1.93±0.01 | 0.001±0.001 | -3.43±2.18 | 0.032 |
|  | Co-Co | 9.16±1.23 | 2.90±0.02 | 0.010±0.003 |  |  |
| CoPc | Co-N | 3.78±0.73 | 1.83±0.12 | 0.002±0.002 | -6.38±0.42 | 0.012 |
|  | Co-C | 2.64±0.34 | 2.33±0.32 | 0.004±0.001 |  |  |
| TTA-TPH-CuCo | Co-N | 2.13±0.13 | 2.04±0.12 | 0.005±0.002 | 5.72±0.85 | 0.015 |
|  | Co-O | 1.78±0.36 | 2.19±0.22 | 0.001±0.001 |  |  |

*^a^CN*, coordination number; *^b^R*, distance between absorber and backscatter atoms; *^c^σ*^2^, Debye-Waller factor to account for both thermal and structural disorders; *^d^ΔE*_0_, inner potential correction; *R* factor indicates the goodness of the fit.

**Table S4**. Comparison of electrocatalytic NO_3_RR performance over the recently reported electrocatalysts.

| **Catalyst** | **NO_3_^−^**  **Concentration** | **Potential**  **(vs. RHE)** | **FE**  **(%)** | **NH_3_ yield**  **(mg·h^-1^ cm^-2^·)** | **Ref** |
| --- | --- | --- | --- | --- | --- |
| **TTA-TPH-CuCo** | **0.1 M** | **−0.75 V** | **92.31** | **13.49** | **This work** |
|  | **0.3 M** |  | **92.16** | **20.80** |  |
|  | **0.5 M** |  | **84.35** | **21.38** |  |
| Fe/Cu-HNG | 0.5 M | −0.3 V | 92.51 | 1.25 | ^[1]^ |
| Mo_2_CT_x_: Fe | 0.1 M | −0.6 V | 70 | 0.219 | ^[2]^ |
| Ag_1.5_Co/CC | 0.1 M | −0.75 V | 82.96 | 3.995 | ^[3]^ |
| CoCu/TiO_2_/Sb_2_Se_3_ | 0.1 M | −0.3 V | 85 | 0.27 | ^[4]^ |
| LF_0.9_Cu_0.1_ | 50 ppm | −0.9 V | 48 | 0.188 | ^[5]^ |
| ISSA In-Pdene | 0.1 M | −0.6 V | 87.2 | 1.12 | ^[6]^ |
| FePc/TiO_2_ | 0.5 M | −0.65 V | 85 | 14 | ^[7]^ |
| COF-366-Fe | 0.1 M | −1.05 V | 85.4 | 2.88 | ^[8]^ |
| NiPr-TPA-COF | 0.1 M | −0.766 V | 80 | 2.5 | ^[9]^ |
| TpBpy-Cu-F | 0.1 M | −0.74 V | 85.4 | 3.77 | ^[10]^ |
| CuCoSP | 0.1 M | −0.175 V | 90.6 | 19.89 | ^[11]^ |
| CuCo NW | 2000 ppm | 300 mA·cm^−2^  (constant current) | 91.7 | 21.76 | ^[12]^ |
| CuCoAl LDH/Vulcan | 0.05 M | −0.8 V | 99.5 | 3.205 | ^[13]^ |

**References**

[1] S. Zhang, J. Wu, M. Zheng, X. Jin, Z. Shen, Z. Li, Y. Wang, Q. Wang, X. Wang, H. Wei, *Nat. Commun.* **2023**, *14*, 3634.

[2] D. F. Abbott, Y. Z. Xu, D. A. Kuznetsov, P. Kumar, C. R. Müller, A. Fedorov, V. Mougel, *Angew. Chem.* **2023**, *135*, e202313746.

[3] Z. Fan, C. Cao, X. Yang, W. Yuan, F. Qin, Y. Hu, X. Sun, G. Liu, Y. Tian, L. Xu, *Angew. Chem.* **2024**, *136*, e202410356.

[4] S. Ren, R. T. Gao, N. T. Nguyen, L. Wang, *Angew. Chem. Int. Ed.* **2024**, *63*, e202317414.

[5] K. Chu, W. Zong, G. Xue, H. Guo, J. Qin, H. Zhu, N. Zhang, Z. Tian, H. Dong, Y.-E. Miao, *J. Am. Chem. Soc.* **2023**, *145*, 21387-21396.

[6] M. Xie, S. Tang, Z. Li, M. Wang, Z. Jin, P. Li, X. Zhan, H. Zhou, G. Yu, *J. Am. Chem. Soc.* **2023**, *145*, 13957-13967.

[7] R. Zhang, C. Li, H. Cui, Y. Wang, S. Zhang, P. Li, Y. Hou, Y. Guo, G. Liang, Z. Huang, *Nat. Commun.* **2023**, *14*, 8036.

[8] H. Hu, R. Miao, F. Yang, F. Duan, H. Zhu, Y. Hu, M. Du, S. Lu, *Adv. Energy Mater.* **2024**, *14*, 2302608.

[9] F. Lv, M. Sun, Y. Hu, J. Xu, W. Huang, N. Han, B. Huang, Y. Li, *Energy Environ. Sci.* **2023**, *16*, 201-209.

[10] Y. Zhu, H. Duan, C. G. Gruber, W. Qu, H. Zhang, Z. Wang, J. Zhong, X. Zhang, L. Han, D. Cheng, *Angew. Chem. Int. Ed.* **2025**, 64, e202421821.

[11] W. He, J. Zhang, S. Dieckhöfer, S. Varhade, A. C. Brix, A. Lielpetere, S. Seisel, J. R. Junqueira, W. Schuhmann, *Nat. Commun.* **2022**, *13*, 1129.

[12] K. Zhang, P. Sun, Y. Huang, M. Tang, X. Zou, Z. Pan, X. Huo, J. Wu, C. Lin, Z. Sun, *Adv. Funct. Mater.* **2024**, *34*, 2405179.

[13] W. Wang, J. Chen, E. C. Tse, *J. Am. Chem. Soc.* **2023**, *145*, 26678-26687.
